# Genomic Prediction in Tetraploid Ryegrass Using Allele Frequencies Based on Genotyping by Sequencing

**DOI:** 10.3389/fpls.2018.01165

**Published:** 2018-08-15

**Authors:** Xiangyu Guo, Fabio Cericola, Dario Fè, Morten G. Pedersen, Ingo Lenk, Christian S. Jensen, Just Jensen, Lucas L. Janss

**Affiliations:** ^1^Center for Quantitative Genetics and Genomics, Department of Molecular Biology and Genetics, Aarhus University, Aarhus, Denmark; ^2^Rijk Zwaan B.V., De Lier, Netherlands; ^3^Research Division, DLF Seeds A/S, Store Heddinge, Denmark

**Keywords:** ryegrass, tetraploid, genomic prediction, genotyping-by-sequencing, sequencing depth

## Abstract

Perennial ryegrass is an outbreeding forage species and is one of the most widely used forage grasses in temperate regions. The aim of this study was to investigate the possibility of implementing genomic prediction in tetraploid perennial ryegrass, to study the effects of different sequencing depth when using genotyping-by-sequencing (GBS), and to determine optimal number of single-nucleotide polymorphism (SNP) markers and sequencing depth for GBS data when applied in tetraploids. A total of 1,515 F_2_ tetraploid ryegrass families were included in the study and phenotypes and genotypes were scored on family-pools. The traits considered were dry matter yield (DM), rust resistance (RUST), and heading date (HD). The genomic information was obtained in the form of allele frequencies of pooled family samples using GBS. Different SNP filtering strategies were designed. The strategies included filtering out SNPs having low average depth (FILTLOW), having high average depth (FILTHIGH), and having both low average and high average depth (FILTBOTH). In addition, SNPs were kept randomly with different data sizes (RAN). The accuracy of genomic prediction was evaluated by using a “leave single F_2_ family out” cross validation scheme, and the predictive ability and bias were assessed by correlating phenotypes corrected for fixed effects with predicted additive breeding values. Among all the filtering scenarios, the highest estimates for genomic heritability of family means were 0.45, 0.74, and 0.73 for DM, HD and RUST, respectively. The predictive ability generally increased as the number of SNPs included in the analysis increased. The highest predictive ability for DM was 0.34 (137,191 SNPs having average depth higher than 10), for HD was 0.77 (185,297 SNPs having average depth lower than 60), and for RUST was 0.55 (188,832 SNPs having average depth higher than 1). Genomic prediction can help to optimize the breeding of tetraploid ryegrass. GBS data including about 80–100 K SNPs are needed for accurate prediction of additive breeding values in tetraploid ryegrass. Using only SNPs with sequencing depth between 10 and 20 gave highest predictive ability, and showed the potential to obtain accurate prediction from medium-low coverage GBS in tetraploids.

## Introduction

Perennial ryegrass (*Lolium perenne* L.) is one of the most widely sown forage grasses in temperate regions (Humphreys, 2005). Low production costs and the perennial character provide high agronomic value, and it is widely used for feeding ruminants (Jensen et al., 2001). The popularity of cultivating perennial ryegrass is mainly due to its re-growth capacity after defoliation and its palatability, digestibility, and nutrient content as feed for ruminants compared with other forage species (Wilkins, 1991).

Compared to diploid ryegrass, the tillers and seed heads of tetraploid ryegrass are longer and the leaves are wider. Tetraploid ryegrass is more open and more prone to wear, but is less susceptible to snow mold and has a better drought tolerance, leading to better performance under continental conditions with frequent dry periods. Palatability and digestibility are better in tetraploid varieties than in diploid varieties, and tetraploids perform better than diploids during grazing (Wilkins, 1991) and lead to a higher animal production (Lantinga and Groot, 1996; O'Donovan and Delaby, 2005).

Perennial ryegrass is an allogamous species (Cornish et al., 1979) due to a gametophytic self-incompatibility system (Cornish et al., 1979). For this reason, it is generally bred, maintained and commercialized as heterogeneous families. Evaluation of F_2_ families is frequently used in breeding programs for outcrossing species such as perennial ryegrass. An F_2_ family consists of the offspring from random interbreeding a full-sib F_1_ family, which are the offspring from an initial bi-parental cross. F_2_ families are evaluated in plot experiments over several locations and years to obtain measurements on yield, agronomic traits, and disease resistance.

Perennial ryegrass breeding has mainly relied on prediction of genetic merit using phenotypic information only (Conaghan and Casler, 2011; Hayes et al., 2013). Using this system, relevant improvements for yield and quality-related traits have been achieved (Wilkins and Humphreys, 2003; McDonagh et al., 2016). However, compared with traits such as rust resistance and spring growth, gains for yield traits like dry matter and seed yield were not as high as expected (Sampoux et al., 2011). In addition, phenotypic selection is costly and time consuming, needing up to 10 years to complete a selection cycle (Wilkins and Humphreys, 2003; Lin et al., 2016). In recent decades, the development of marker technology allowed adoption of genomic prediction (GP) strategies, which have been highly beneficial and led to a reduction of cost in practical animal and plant breeding programs (Hickey et al., 2017). In GP, dense markers distributed across the whole genome can be used simultaneously to predict breeding values (Meuwissen et al., 2001). The quantitative trait loci (QTLs) affecting the traits of interest are assumed in linkage disequilibrium (LD) with one or more single-nucleotide polymorphism (SNP) markers. Thus, a sufficiently dense and well-distributed set of markers allows all QTLs to be in LD with at least one marker, and this LD can be exploited in GP to ensure accurate prediction of breeding values as a basis for selection decisions.

The prospects for implementing GP in forage grass breeding were recently reviewed by Hayes et al. (2013). Several GP studies have been reported for crops such as maize and wheat (Crossa et al., 2010, 2014), and the first investigations in diploid perennial ryegrass also demonstrated great potential for using GP (Fè et al., 2015a, 2016). However, GP studies for tetraploid ryegrass, to our knowledge, have not yet been carried out. The implementation of GP in tetraploid ryegrass may be more challenging than in diploid ryegrass, because families of tetraploids will show a higher heterozygosity than families of diploids. This may hamper accurate estimation of genomic relationships and genomic breeding values.

Genotyping-by-sequencing (GBS) was developed by Elshire et al. (2011) as a robust genotyping approach. GBS uses methylation sensitive restriction enzymes to reduce genome complexity. GBS is a good approach to estimate genome-wide allele frequency profiles in pooled samples for outbred heterogeneous varieties (Byrne et al., 2013). Moreover, for association studies and GP studies, calling of genotypes can be avoided by directly using allele frequencies from GBS, which facilitates measurements on family pools (Ashraf et al., 2014). Use of GBS data also poses some challenges; in particular, sequencing depth needs to be optimized carefully. At low depth, genotyping errors and missing values are an issue (Poland and Rife, 2012), and result in biased estimates of allele-effect and heritability (Ashraf et al., 2014, 2016). At higher sequencing depth the accuracy of genotype estimates is improved (Sims et al., 2014), but under a fixed budget, the number of samples that can be sequenced would be reduced, which reduces power of the entire experiment (Ashraf et al., 2014). Several investigations on how sequencing depth affects association studies and estimation of genomic heritability have been conducted (Garner, 2011; Sims et al., 2014; Ashraf et al., 2016). As reviewed by Poland and Rife (2012), GBS has become a flexible and low cost tool for plant genetics and breeding. It has been demonstrated that GBS can effectively generate high-density genome wide markers in a range of species (Elshire et al., 2011; Poland and Rife, 2012; Poland et al., 2012; Beissinger et al., 2013; Crossa et al., 2013; Zhang et al., 2015; Fè et al., 2016; Cericola et al., 2018). With GBS, an accurate GP model was derived for the large, complex, and polyploid wheat genome (Poland et al., 2012). In addition, GBS also has been applied on diploid ryegrass for genomic prediction (Fè et al., 2015a, 2016). However, to our knowledge the optimization of sequencing depth for GP in tetraploid ryegrass has not been reported yet.

The aims of this study were: (1) to investigate the possibility of implementing genomic prediction in tetraploid perennial ryegrass, (2) to study the effects of different sequencing depth when using GBS, and (3) to determine the optimal number of SNPs to include in genomic prediction when GBS are applied in tetraploid ryegrass.

## Materials and methods

### Plant material

Both phenotype and genotype data were derived from 1,515 F_2_ families from a commercial breeding program from DLF A/S, Denmark. F_2_ families originated from a pair-cross between two parents; F_1_ seeds from both parent plants were pooled; F_1_ families were sown in small protected plots to cross-fertilize; and finally F_2_ seeds were harvested and used for field-testing of F_2_ families. A detailed description of testing procedures was provided by Fè et al. (2015b).

Phenotypic records, defined below, consisted of historical data from F_2_ families, which were sown between 2004 and 2016 at 8 locations in Denmark, the Netherlands, France, and United Kingdom, and cultivated according to the local management schemes. In all locations, F_2_ families were tested in trials including 12 families in a randomized experiment with two replicates for each family. Details of testing and recording procedures were the same as for diploid ryegrass as described previously (Fè et al., 2015b). The dataset analyzed included records of three traits:

1. Dry matter yield (DM), expressed in kg/m^2^ and obtained from multiple cuts over 2 years. For analyses, the total yield during the first year and the total yield during the second year were used so that each family had yield measurements from two years; to validate genomic predictions, the average yield of the two years was predicted.
2. Heading date (HD), defined as the day on which spikes are visible over the general plots, and expressed in days since January 1st. HD was scored in plots for seed multiplication, which were farmed for one cropping season only.
3. Rust resistance (RUST), measured during the period of maximum infection, both in regular yield plots, and in mini plots, which were cultivated only for 1 year. The level of infection was determined by visual scoring from 1 (plants completely covered by rust) to 9 (no sign of rust infection). Plots were cut between the different scoring time points to make the scores independent.

Descriptive statistics including mean value, standard deviation, minimum, maximum, number of families, number of records, number of plots, and number of sowing year × location × management levels are listed in Table 1.

**Table 1:** Descriptive statistics^a^ for three traits.

**Trait^b^**	**No. Fam**	**No. Rec**	**No. Plot**	**No. YLM**	**Mean**	***SD***	**Min**	**Max**
DM	1,188	5,312	3,414	27	1.33	0.37	0.41	2.5
HD	979	1,810	1,810	7	155.64	7.51	136	178
RUST	1,506	13,545	5,368	22	5.64	1.99	1	9

a *No. Fam, number of families; No. Rec, number of records; No. Plot, number of plots; No. YLM, number of sowing year × location × management levels; Mean, mean value; SD, standard deviation; Min, minimum value; Max, maximum value*.

b *DM, dry matter yield; HD, heading date; RUST, rust resistant*.

### Filtering of GBS data and calculation of allele frequencies for each family

**Figure 1:**
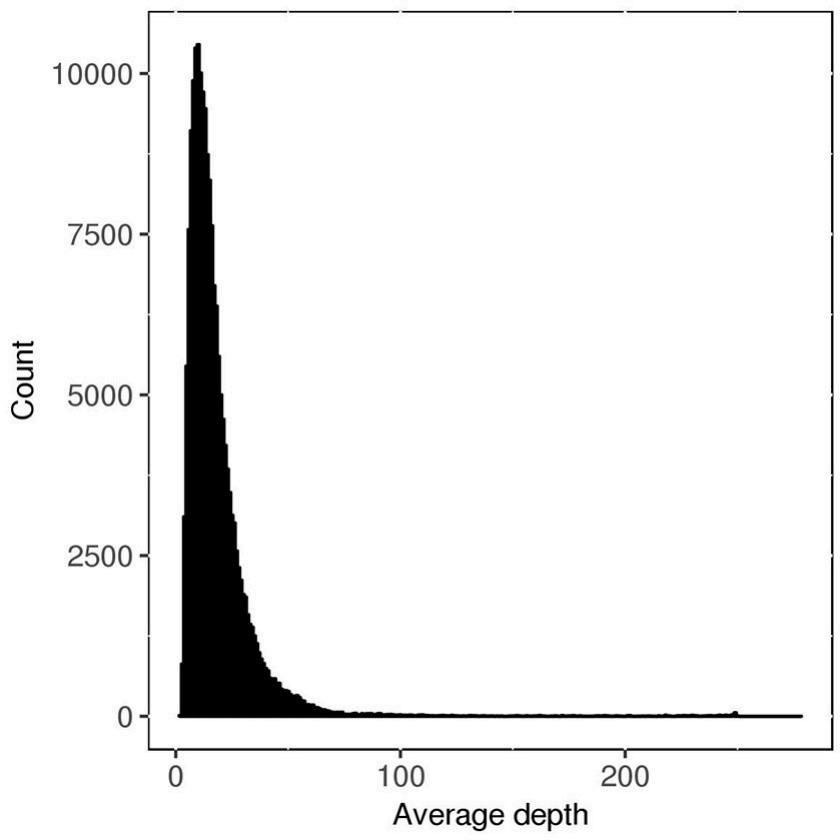
Distribution of average read depth for each SNP before filtering by different strategies.

Genotypic data was produced as described previously (Fè et al., 2015a). In total, 1,515 F_2_ families were sequenced. A total of 51 libraries were prepared, with up to 96 families per library. Each library was sequenced on multiple lanes of an Illumina HiSeq2000 (single-end). On average, 12.9 million 100 bp single-end reads were produced per sample. A draft sequence assembly (Byrne et al., 2015) was used for the alignment of data for each family, and initially 18.6 million SNPs were identified. A first, quite liberal, filtering of the raw SNP data was performed by removing: (1) SNPs with missing rate higher than 50%; (2) SNPs with allele frequencies lower than 0.01 or higher than 0.99; (3) SNPs with average read depth smaller than 1. This left 188,832 SNPs available for our analysis, which included further, more stringent, filtering steps for the SNPs. The average read depth for the 188,832 SNPs ranged from 1 to 278, with mean of 19. The distribution of average read depth for each SNP is shown in Figure 1.

Differently from SNP chip data, where genotypes are explicitly called, the genotype of a SNP is obtained here in the form of an allele frequency ($\hat{g_{ij}}$), which is estimated as the ratio between number of reads for alternative allele (*S*_1_*ij*__) and the total number of reads (*S*_*T*_*ij*__), which is the sum of number of reads for the reference allele (*S*_2_*ij*__) and *S*_1_*ij*__, for each sample:

$$\begin{array}{l} \hat{g_{ij}}=\frac{S_{1_{ij}}}{S_{T_{ij}}}=\frac{S_{1_{ij}}}{S_{1_{ij}}+\;S_{2_{ij}}}. \end{array}$$

### SNP filtering strategies

**Figure 2:**
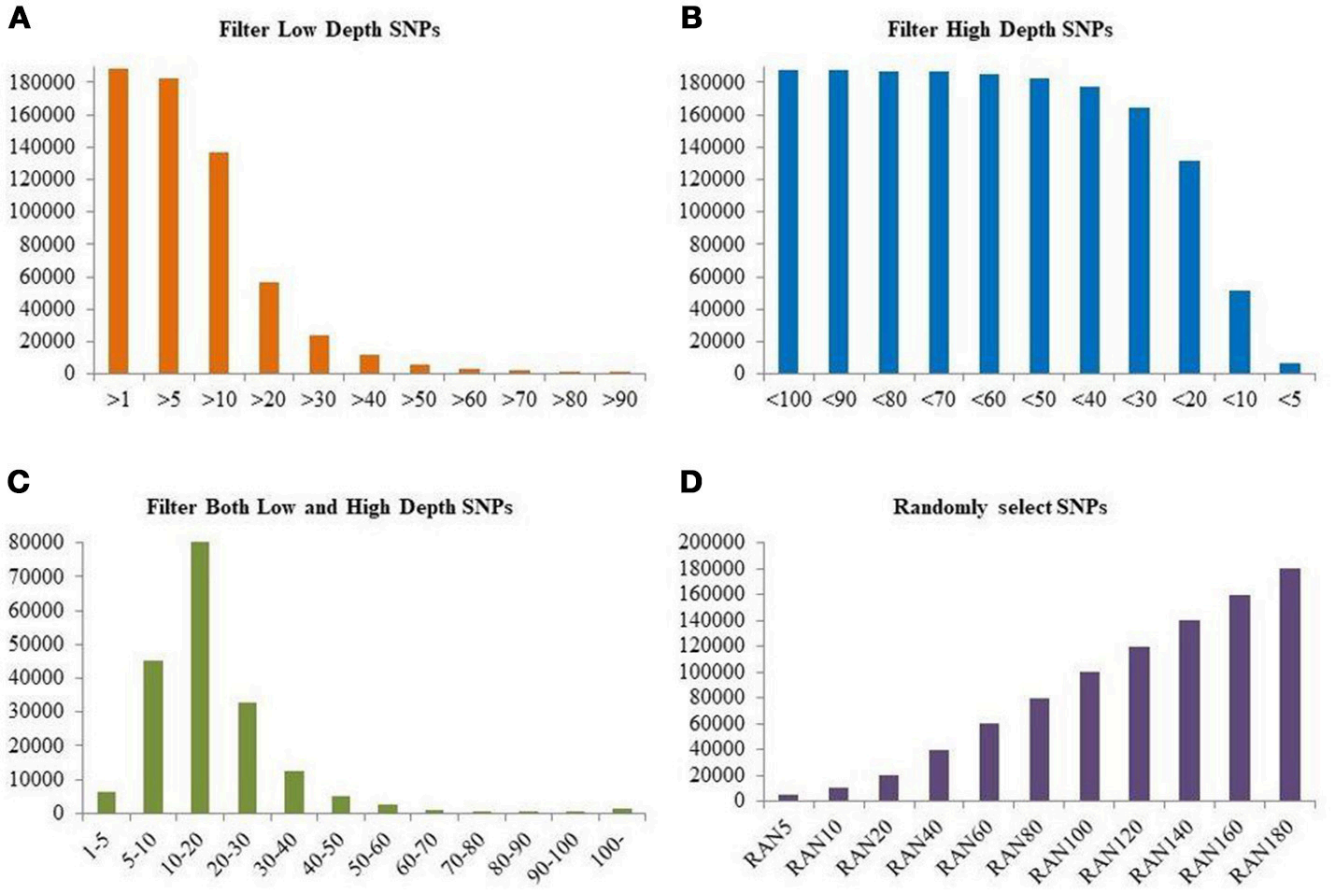
Numbers of SNPs retained for four different filtering strategies. **(A)** Number of SNPs in each FILTLOW dataset with different lower threshold; **(B)** Number of SNPs in each FILTHIGH dataset with different upper threshold; **(C)** Number of SNPs in each FILTBOTH dataset with different lower and upper thresholds; **(D)** Number of SNPs in each RAN dataset with different size. The y-axis is the number of SNPs after filtering. In **(A–C)**, the x-axis is different subsets of SNPs with various sequencing depth, and the x-axis in **(D)** is randomly selected subset with different size.

In order to study the effect of sequencing depth of GBS data, additional SNP filtering was performed. First, SNPs having average depth lower than a certain value were filtered out in 11 levels, with minimum depth from 1 to 90 (FILTLOW1 to FILTLOW11); second, SNPs having average depth higher than a certain value were filtered out in 11 levels, with maximum depth from 100 to 5 (FILTHIGH1 to FILTHIGH11); third, SNPs having average depth outside a certain range were filtered out (equivalent to keeping SNPs with average depth within that range), using 12 different ranges (FILTBOTH1 to FILTBOTH12); finally, SNPs were kept randomly with 11 different data sizes from 5 to 180 k (RAN5 to RAN180), and repeated for 10 times. In summary, there were four filtering strategies, FILTLOW, FILTHIGH, FILTBOTH, and RAN, and the number of scenarios was, respectively, 11, 11, 12, and 11, where the latter (RAN) was repeated 10 times. A summary of SNPs used in each filtering scenario is shown in Figure 2 and the details are shown in Supplementary Table 1.

### Statistical model and methods

A single trait model was used to estimate variance components and fixed effects, and to predict breeding values as well as other random effects in the model:

$$y=Xb+Z_{g}g+Z_{a}a+Z_{p}p+Z_{i_{1}}i_{1}+Z_{i_{2}}i_{2}+e,$$

in which **y** was the vector of phenotypic values of the trait DM, HD or RUST; **b** was the vector of fixed effects (sowing year × location × management × trial × farming year); **g** was the vector of additive genomic family effects; **a** was the vector of residual genetic family effects which were not explained by the genomic information; **p** was the vector of random plot effects; **i**_1_ and **i**_2_ were vectors of genotype by environment (G×E) effects [**i**_1_: family × sowing year × location × management (GSLM), **i**_2_: family × sowing year × location × management × farming year (GSLMF)]; and **e** was the vector of random residual effects. **X**, **Z**_g_, **Z**_a_, **Z**_p_, **Z**_**i**_1__, and **Z**_**i**_2__were incidence matrices associating **b**, **g**, **a**, **p**, **i**_1_, and **i**_2_ with **y**. The random effects were assumed to be independent of each other and normally distributed, that is, $g\text{​​​}\sim \text{​​​}N(0,G^{*}\sigma_{g}^{2}),a\text{​​​}\sim \text{​​​}N(0,I\sigma_{a}^{2}),p\text{​​​}\sim \text{​​​}N(0,I\sigma_{p}^{2}),i_{1}\text{​​​}\sim \text{​​​}N(0,I\sigma_{i_{1}}^{2}),i_{2}\text{​​​}\sim \text{​​​}N(0,I\sigma_{i_{2}}^{2}),e\text{​​​}\sim \text{​​​}N(0,I\sigma_{e}^{2})$, in which G^*^ was the corrected **G** matrix of additive genomic relationships constructed based on the genomic information, **I** was the identity matrix, and $\sigma_{g}^{2}$, $\sigma_{a}^{2}$, $\sigma_{p}^{2}$, $\sigma_{i_{1}}^{2}$, $\sigma_{i_{2}}^{2}$, and $\sigma_{e}^{2}$ were the variances of additive genomic effects, residual genetic effects, random plot effects, first genetic by environment effects, second genetic by environment effects, and residuals, respectively. For DM and RUST, the general model was applied in the analysis, while for HD, the effects of **p** and **i**_2_ were excluded since there was only one score and only one environment in each family for HD.

The method to compute the **G** matrix was based on a modification of VanRaden (2008) to use allele frequencies (ranging between 0 and 1) instead of SNP genotype calls. A matrix (**F**) with allele frequencies for each sample was centered by the mean SNP frequencies to create matrix **M** (${\text{M}}_{\text{j}}={\text{F}}_{\text{j}}-\bar{{\text{F}}_{\text{j}}}$). Then, the **G** matrix was obtained by computing **M** multiplied by its own transpose and scaled by the sum of expected SNP variances across genotypes (**G** = **MM**′/**K**). The scale parameter used for tetraploid F_2_ families is half that used for diploid F_2_ families as computed by Ashraf et al. (2014) and as applied in the study by Fè et al. (2015a), because the number of alleles in F_2_ family pools is eight for tetraploid families, which is double that of diploid families:

$$\begin{array}{l} \text{K}=0.125\sum \bar{F_{j}}\left(1-\bar{F_{j}}\right). \end{array}$$

Finally, the **G** matrix was corrected for the extra binomial variance due to limited sequencing depth. The correction was derived by Cericola et al. (2018) and simply can be done according to ploidy number and the average depth of the sample. Corrected **G** matrix (G^*^) was calculated by scaling down the diagonal elements of each individual as follows:

$$\begin{array}{l} D_{c_{i}}=D_{b_{i}}\left(1-\frac{n-1}{\bar{S_{T_{i}}}+n-1}\right), \end{array}$$

where *D*_*b*_*i*__ is the *ith* element of the biased diagonal element in **G** and *D*_*c*_*i*__ is the corrected diagonal element in G^*^, $\bar{S_{T_{i}}}$ is the average *S*_*T*_*ij*__ for each individual across all SNPs, and *n* is the ploidy number, which is eight as mentioned before.

For each of the four filtering scenarios, single trait analyses were run on the subsets of SNPs, which were previously created according to different filtering strategies (Figure 2 and Supplementary Table 1). Variance components and their standard errors (SE) were estimated by restricted maximum likelihood (REML) using the DMU software package (Madsen and Jensen, 2013).

The phenotypic variance of family means was calculated as the sum of weighted variance components:

$$\begin{array}{l} \sigma_{P_{f}}^{2}=\bar{G^{*}}\sigma_{g}^{2}+\sigma_{a}^{2}+\sigma_{p}^{2}/n_{p}+\sigma_{i_{1}}^{2}/n_{i_{1}}+\sigma_{i_{2}}^{2}/n_{i_{2}}+\sigma_{e}^{2}/n_{e}, \end{array}$$

where $\bar{G^{*}}$ is the average diagonal of the corrected genomic relationship matrix (G^*^ matrix), *n*_*p*_ is the average number of plots for each family, *n*_*i*_1__ and *n*_*i*_2__ are the average numbers of environments for each family, and *n*_*e*_ is average number of replicates across all fields for each family. Accordingly, genomic family heritability based on multiple plots was calculated as $h_{f}^{2}=\frac{\bar{G^{*}}\sigma_{g}^{2}}{\sigma_{P_{f}}^{2}}$. To evaluate importance of each random effect in the model, phenotypic variance of a single plot was also calculated:

$$\begin{array}{l} \sigma_{P_{p}}^{2}=\bar{G^{*}}\sigma_{g}^{2}+\sigma_{a}^{2}+\sigma_{p}^{2}+\sigma_{i_{1}}^{2}+\sigma_{i_{2}}^{2}+\sigma_{e}^{2}. \end{array}$$

In the calculation of $\sigma_{P_{f}}^{2}$, $\sigma_{P_{p}}^{2}$ and $h_{f}^{2}$, $\sigma_{p}^{2}$, and $\sigma_{i_{2}}^{2}$ were not considered for HD due to the reduced recording strategy for this trait. This was used to compute the relative contribution of each random effect to the total phenotypic plot variance.

### Cross-validation

To estimate the accuracy of genomic breeding values (GEBVs), a leave-one-family-out cross-validation (CV) strategy was applied. In each CV round, the phenotypes of one family were masked and then all other families were used to train the prediction model and to predict the family with phenotypes masked.

Before CV, the whole dataset was used to estimate variance components and fixed effects. Corrected phenotypes (*y*_*c*_) were computed by subtracting the estimates of the fixed effects. Predictive ability was measured as $cor\left(\bar{y_{c}},ĝ\right)$, which is theoretically not larger than the square root of $h_{f}^{2}$ (Legarra et al., 2008) because breeding values predict genetic effects and not environment. $\bar{y_{c}}$ is the average *y*_*c*_ for each family. Furthermore, to assess bias of predictions, regression coefficient of $\bar{y_{c}}$ on ĝ was calculated. The deviation of this regression coefficient from 1 represents the level of bias.

## Results

**Figure 3:**
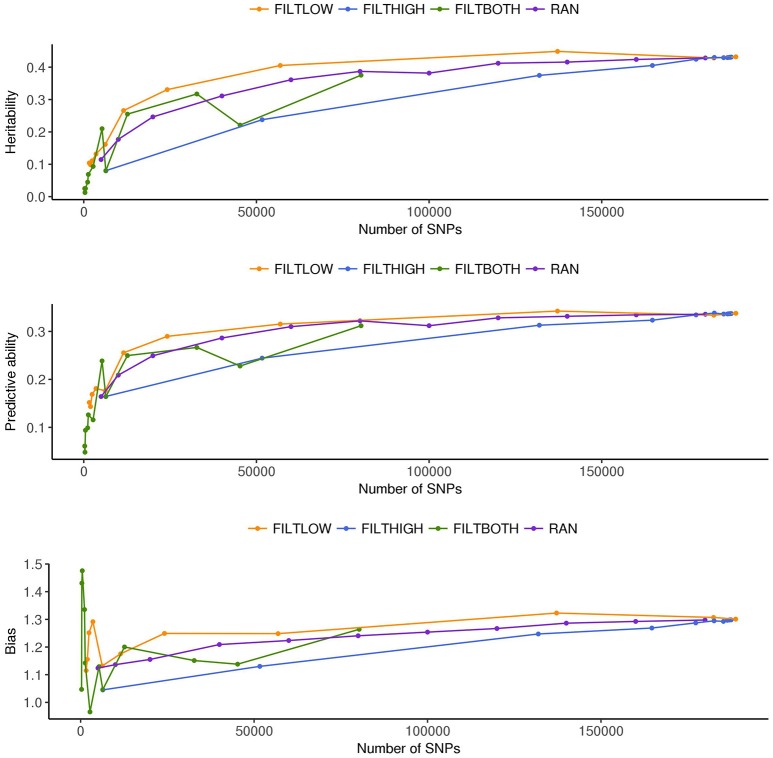
Estimated heritability, predictive ability and bias in different SNP filtering scenarios^1^ for dry matter yield. ^1^ 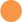
FILTLOW, strategy filtering out SNPs having low average depth; 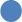
FILTHIGH, strategy filtering out SNPs having high average depth; 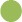
FILTBOTH, strategy filtering out SNPs having both low average and high average depth; 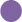
RAN, strategy keeping SNPs randomly with different data size.

**Figure 4:**
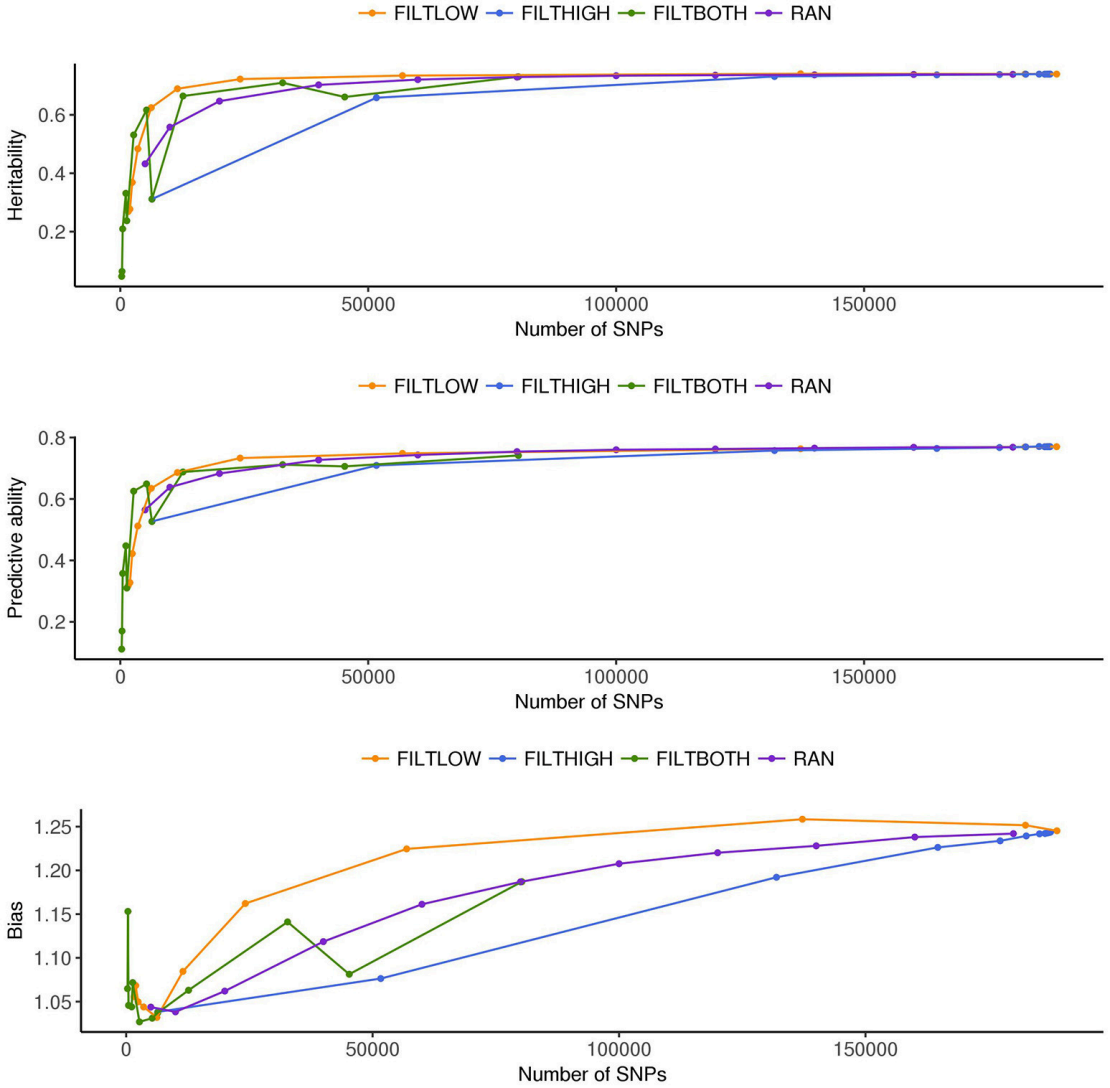
Estimated heritability, predictive ability and bias in different SNP filtering scenarios^1^ for heading date. ^1^ 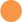
FILTLOW, strategy filtering out SNPs having low average depth; 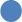
FILTHIGH, strategy filtering out SNPs having high average depth; 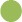
FILTBOTH, strategy filtering out SNPs having both low average and high average depth; 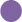
RAN, strategy keeping SNPs randomly with different data size.

**Figure 5:**
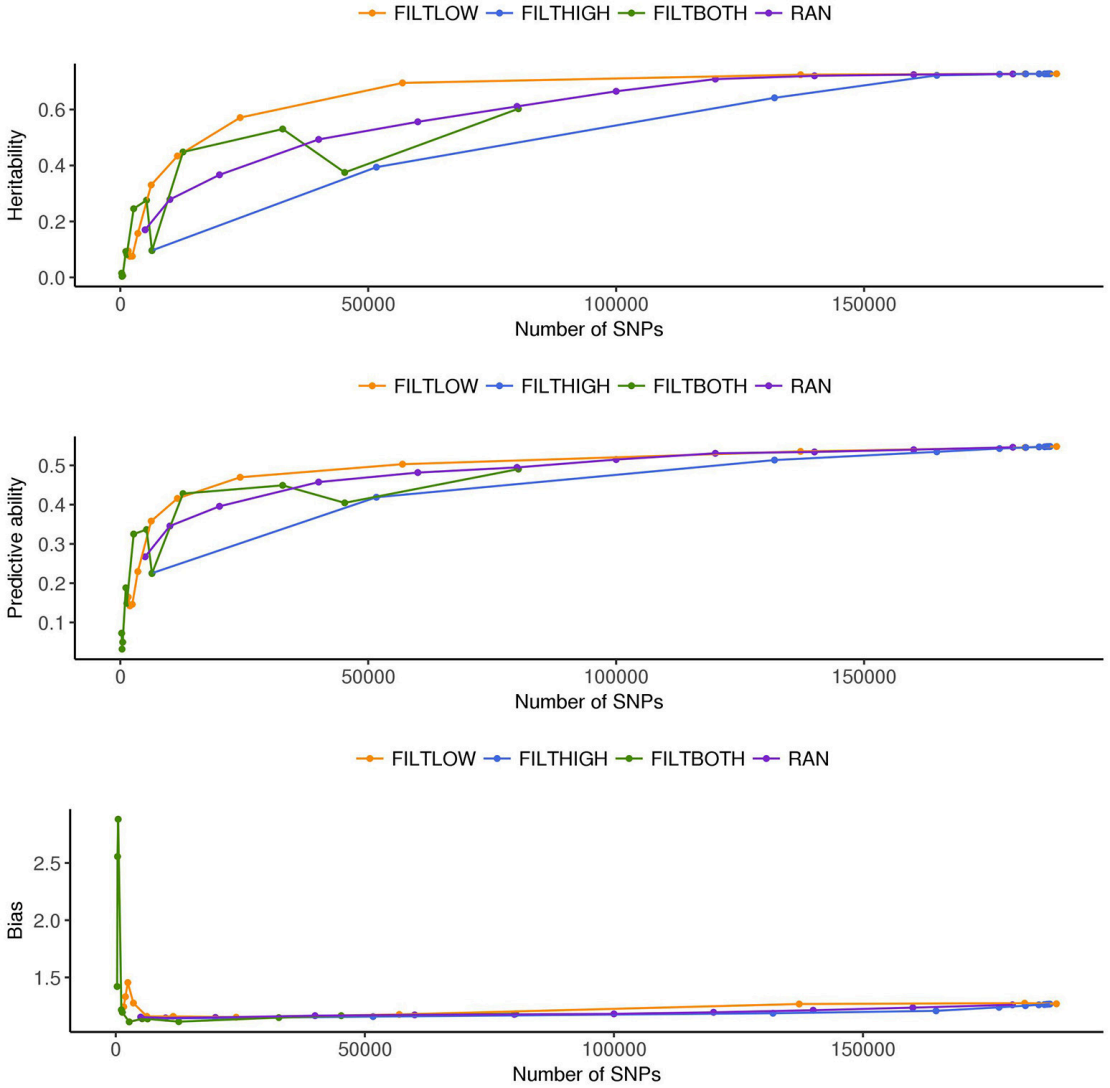
Estimated heritability, predictive ability and bias in different SNP filtering scenarios^1^ for rust resistance. ^1^ 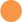
FILTLOW, strategy filtering out SNPs having low average depth; 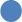
FILTHIGH, strategy filtering out SNPs having high average depth; 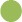
FILTBOTH, strategy filtering out SNPs having both low average and high average depth; 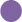
RAN, strategy keeping SNPs randomly with different data size.

**Figure 6:**
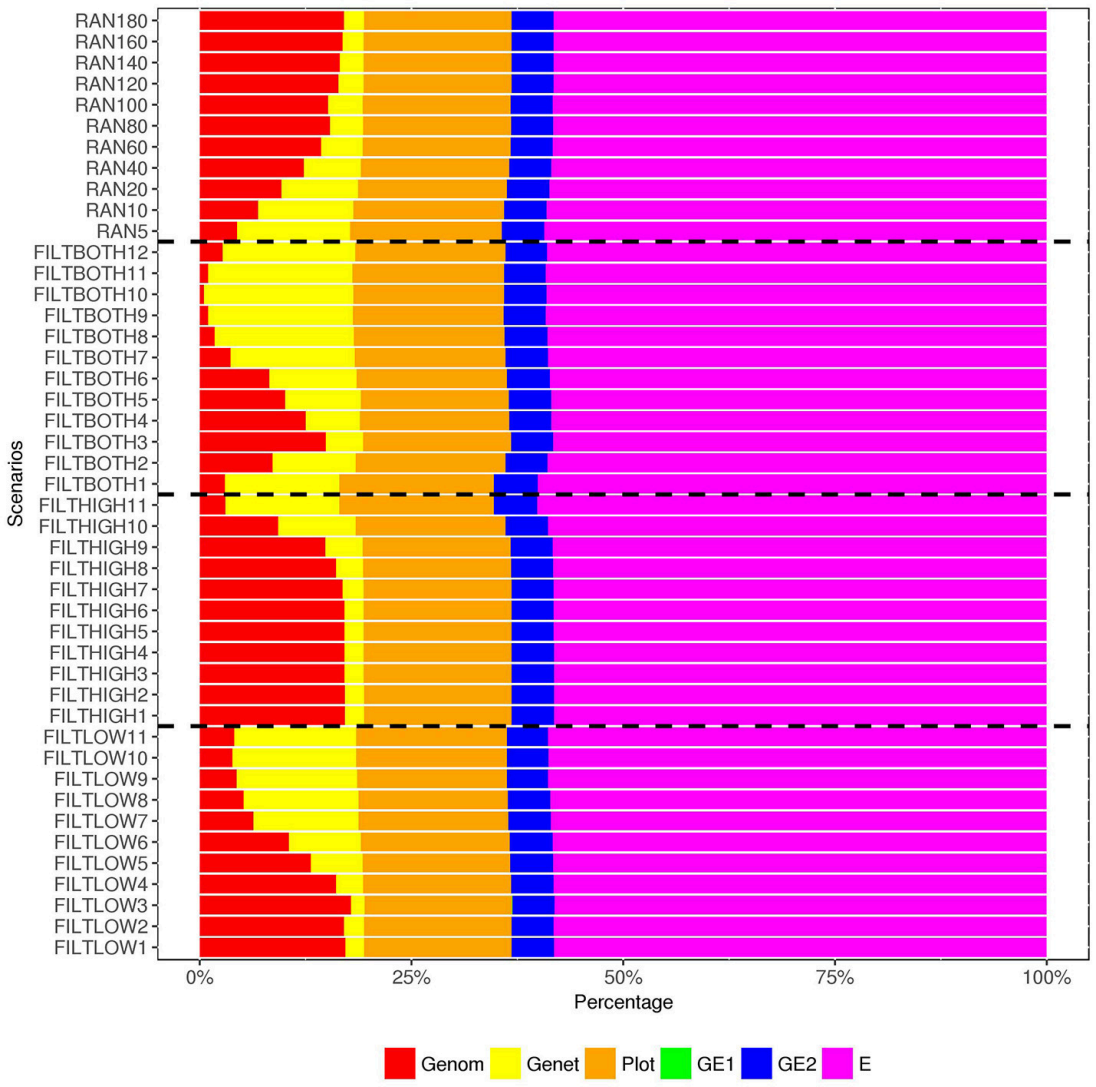
Percentage of variance components^1^, ^2^ over the total phenotypic variance for dry matter yield. ^1^ 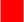
Genom, Additive genomic variance; 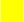
Gene, Residual genetic variance; 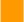
PLOT, random plot variance; 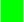
GE1, family × sowing year × location × management variance; 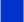
GE2, family × sowing year × location × management × farming year variance; 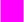
E, residual environment variance. ^2^ GE1 is too small to be visible, so that there are only five variances can be observed in this figure.

**Figure 7:**
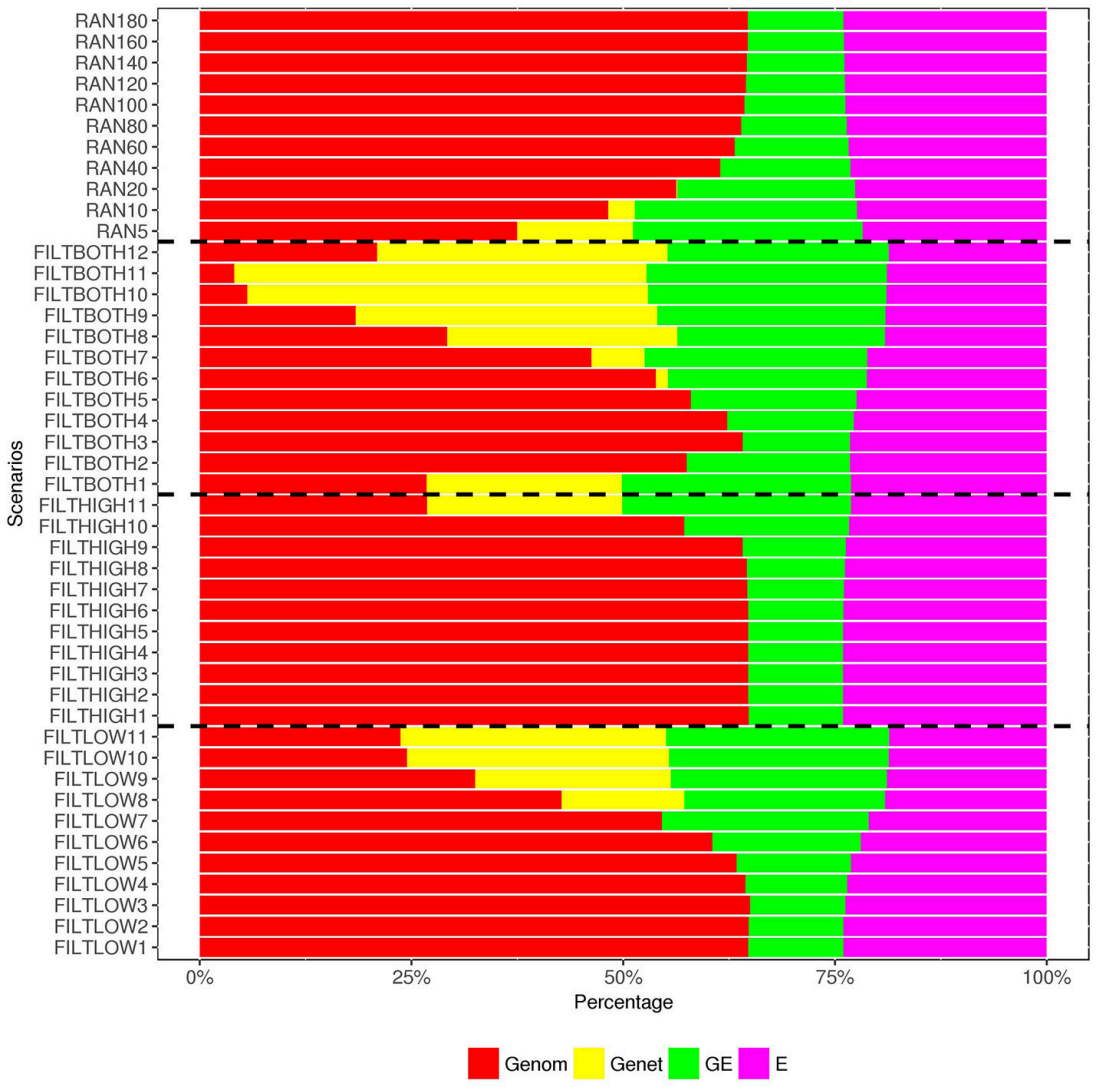
Percentage of variance components^1^ over the total phenotypic variance for heading date. ^1^ 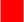
Genom, Additive genomic variance; 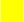
Gene, Residual genetic variance; 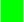
GE, family × sowing year × location × management variance; 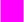
E, residual environment variance.

**Figure 8:**
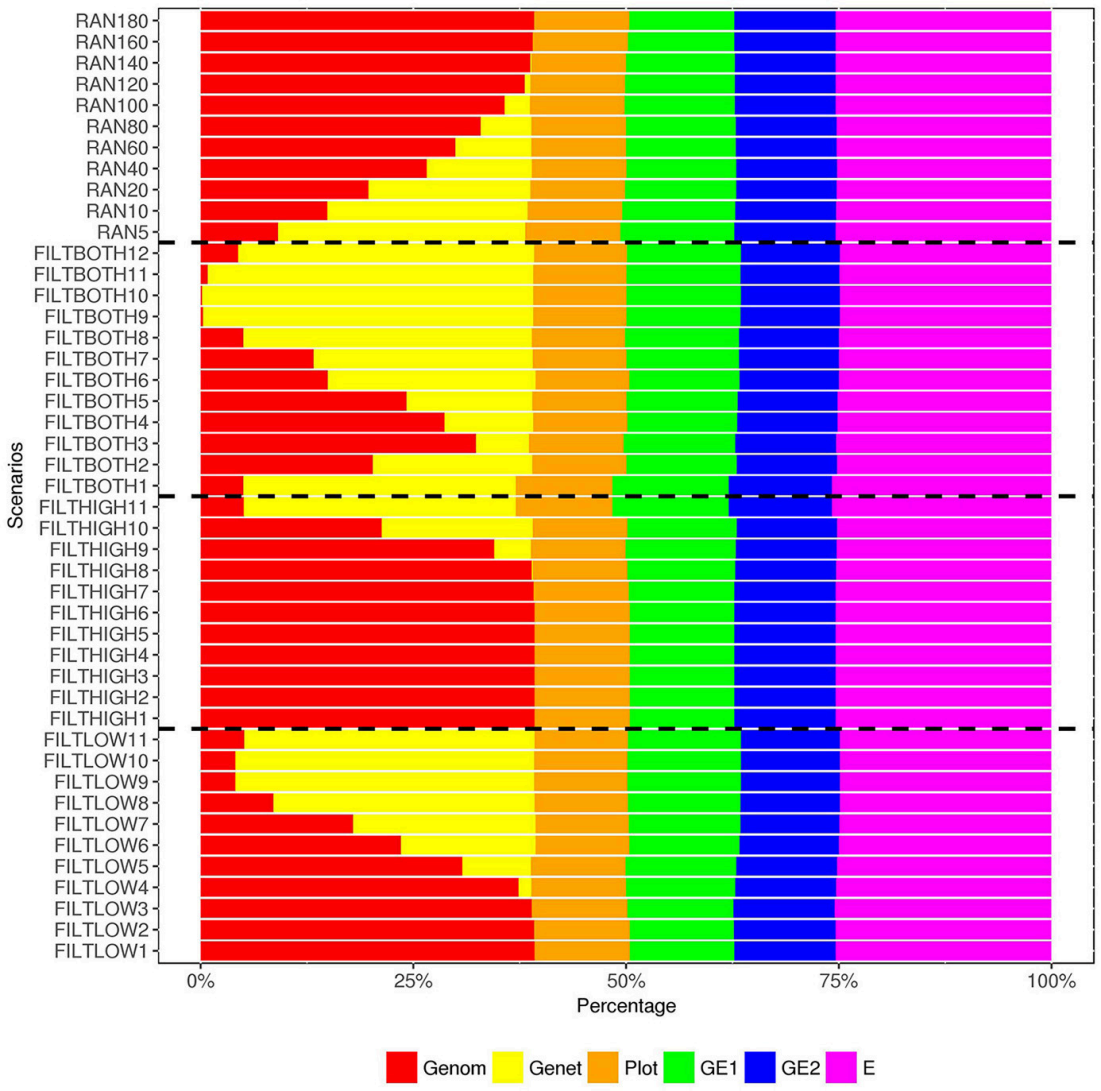
Percentage of variance components^1^ over the total phenotypic variance for rust resistance. ^1^ 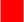
Genom, Additive genomic variance; 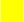
Gene, Residual genetic variance; 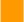
PLOT, random plot variance; 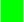
GE1, family × sowing year × location × management variance; 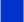
GE2, family × sowing year × location × management × farming year variance; 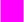
E, residual environment variance.

In order to interpret the results from each scenario, Figures 2–8 were created. Figure 2 and Supplementary Table 1 show the numbers of SNPs retained for four different filtering strategies. Figure 2 shows four bar charts according to the data filtering levels. Figures 3–5 show the estimated heritability, predictive ability and bias in different SNP filtering scenarios for three traits, DM, HD and RUST, respectively. In these figures, line charts were plotted as a function of number of SNPs included in each model. Figures 6–8 show the percentages of explained variance, i.e., each variance components over the total phenotypic variance, for three traits. In these three figures, bar charts were plotted for all scenarios.

### SNP filtering strategies

The first filtering strategy FILTLOW used an increasing lower threshold for average SNP read depth, and the number of SNPs included decreased from 188,832 for FILTLOW1 to 1,587 for FILTLOW11. The second filtering strategy FILTHIGH used a decreasing upper threshold for average SNP read depth, and the number of SNPs included decreased from 187,516 for FILTHIGH1 to 6,389 for FILTHIGH11. In this data, a large proportion of SNPs had read depth between 10 and 20, which caused large reductions in the numbers of SNP when either the lower threshold for read depth increased to 20, or when the upper threshold for read depth decreased to 10. For instance, between FILTLOW >10 and FILTLOW >20, the number of SNPs kept dropped from 73 to 30%, and between FILTHIGH < 20 and FILTHIGH < 10, the number of SNPs kept dropped from 70 to 27%. The SNPs with depth lower than 10 and 5 accounted for 27 and 3% of the full data, respectively. In the third filtering strategy, SNPs were kept in a certain interval of average read depth. The percentage of SNPs kept varied from 43% for FILTBOTH3 having average read depth from 10 to 20, to 0.1% for FILTBOTH11 having average read depth from 90 to 100. In addition to three filtering strategies for average read depth, the RAN filtering strategy kept random subsets from the full dataset, ranging from 5 K (RAN5) to 180 K (RAN180) SNPs; in percentages this corresponds to 3% of SNPs in RAN5 to 95% of SNPs in RAN180.

### Variance components and heritabilities

Figures 3–5 show the effects of different filtering strategies on estimates of $h_{f}^{2}$ for the traits DM, DH, and RUST, respectively. For DM, the highest estimate of $h_{f}^{2}$ was 0.45 (FILTLOW3). In the FILTLOW scenarios, the estimated $h_{f}^{2}$ increased slightly from FILTLOW1 to FILTLOW3, and decreased fast afterwards. In the FILTHIGH scenarios, the estimated $h_{f}^{2}$ generally decreased from FILTHIGH1 to FILTHIGH11, and the decrease was more obvious from FILTHIGH8. In the FILTBOTH scenarios, the estimated $h_{f}^{2}$ was highest in FILTBOTH3, showed a small decrease in FILTBOTH4, and larger reductions in other FILTBOTH scenarios. In the RAN scenarios, the estimated $h_{f}^{2}$ increased along with the number of SNPs included in the model, i.e., increased from RAN5 to RAN180, with rate of increase gradually reducing. For HD, the trends of heritability estimates within the four filtering strategies were the same as for DM, and the highest estimate of $h_{f}^{2}$ was also for FILTLOW3 at 0.74. For RUST, the highest estimate of $h_{f}^{2}$ was 0.73 for FILTHIGH1. In the FILTLOW scenarios, the estimated $h_{f}^{2}$ were similar for FILTLOW1 to FILTLOW3, and also decreased fast afterwards. The trends for the other three filtering strategies (FILTHIGH, FILTBOTH and RAN) were the same as for DM and HD.

In our analysis model, we also include a variance component for residual genetic effects ($\sigma_{a}^{2}$), i.e., the part of genetic effects that cannot be explained by genomic markers. Figures 6–8 show that the percentage of $\sigma_{a}^{2}$ over $\sigma_{P_{p}}^{2}$ changed in the different scenarios. When the number of SNPs increased, the percentage of additive genetic variance explained by markers generally increased while the percentage of residual genetic variance decreased, and the percentage of total genetic variance (sum of $\bar{G^{*}}\sigma_{g}^{2}$ and $\sigma_{a}^{2}$) over $\sigma_{P_{p}}^{2}$ remained relatively similar for all scenarios.

For the variance of plot effects estimated for DM and RUST, the percentages of $\sigma_{p}^{2}$ over $\sigma_{P_{p}}^{2}$ were consistent across all the scenarios but different between DM and RUST. The percentage of variance due to plot effects in DM was about twice as large as the plot variance in RUST (Figure 6, 8). For DM, $\sigma_{p}^{2}$ and total genetic variance had similar magnitude, but for RUST, $\sigma_{p}^{2}$only accounted for 29% of total genetic variance.

As shown in Figures 6, 8, the estimates of variance for G×E interactions GSLM ($\sigma_{i_{1}}^{2}$) and GSLMF ($\sigma_{i_{2}}^{2}$) were similar among all the scenarios, but different in DM and RUST. For DM, estimates of $\sigma_{i_{1}}^{2}$ were not significantly different from 0. However, for RUST, estimates for both $\sigma_{i_{1}}^{2}$ and $\sigma_{i_{2}}^{2}$ were significantly different from 0, with the average percentages of $\sigma_{i_{2}}^{2}$ being slightly larger than$\sigma_{i_{1}}^{2}$. For HD, the estimates of $\sigma_{i_{1}}^{2}$ varied more between scenarios than for DM and RUST, and the percentage of $\sigma_{i_{1}}^{2}$ over $\sigma_{P_{p}}^{2}$ ranged from 11 to 28%. When the number of SNPs increased, this percentage generally decreased. For HD, less phenotypic records were available and therefore variance components were estimated with lower accuracy.

The estimation of residual variance ($\sigma_{e}^{2}$) was generally consistent among all scenarios. The average percentage of $\sigma_{e}^{2}$ was 59, 22, and 25%, for DM, HD and RUST, respectively. The largest difference between residual variance estimates for the different scenarios was 5% for HD (24% in FILTHIGH5 vs. 19% in FILTLOW11), 2% for DM (60% in FILTHIGH11 vs. 58% in FILTLOW3), and 1% in RUST (26% in FILTHIGH11 vs. 25% in FILTLOW11).

Details on estimated variance components and heritabilities, together with their standard errors (SE), for three traits in all F_2_ families are available in Supplementary Table 2 (DM), Supplementary Table 3 (HD) and Supplementary Table 4 (RUST).

### Cross-validation

Detailed results from CV for three traits are available in Supplementary Table 5 (HD).

Figure 3 (DM), Figure 4 (HD), and Figure 5 (RUST), show that the predictive ability generally increased when the number of SNPs included in the analysis increased. The highest predictive ability for DM was provided by dataset FILTLOW3 (0.34) with 137,191 SNPs having average depth higher than 10, the highest predictive ability for HD was provided by dataset FILTHIGH5 (0.77) with 185,297 SNPs having average depth lower than 60, and the highest predictive ability for RUST was provided by dataset FILTLOW1 (0.55) with 188,832 SNPs having average depth higher than 1, which was equivalent to including all markers.

Randomly filtering out SNPs and varying the number of SNPs showed that predictive ability generally increased with increasing number of SNPs included in the analysis. Above 80–100 K SNPs, effects of further increases were limited.

Overall, with an increase in the number of SNPs included in the analysis, the bias, which is the deviation from 1 for the regression of predictions on observed phenotypes, also increased. For all three traits, the FILTLOW strategy showed more biased predictions than RAN, whereas the FILTHIGH strategy showed less biased prediction than RAN. In addition, larger bias was always observed together with better predictive ability. For DM, strategy FILTLOW3 provided best predictive ability, but the bias when using this subset of SNPs was also high (regression coefficient was 1.32). For HD, the most biased prediction was also provided by dataset FILTLOW3 (regression coefficient was 1.26), though the best predictive ability was provided by the dataset FILTHIGH5, the predictive ability when using dataset FILTLOW3 was only 0.01 lower than FILTHIGH5. For RUST, FILTLOW1 provided highest predictive ability, and the bias provided by this dataset was slightly larger (regression coefficient was 1.27) than other scenarios, except for models with few SNPs included.

## Discussion

To investigate the potential for genomic prediction in tetraploid ryegrass we analyzed data from 1,515 F_2_ families. All families were genotyped using GBS with an average sequencing depth of 19. GBS data, with various strategies for filtering SNPs, were used in GP models, and we compared heritabilities and predictive abilities to determine optimal SNP numbers and sequencing depth for genomic prediction in tetraploid ryegrass. Among all the filtering scenarios, the highest estimates for genomic heritability of family means were 0.45, 0.74 and 0.73 for DM, HD and RUST, respectively. The predictive ability generally increased as the number of SNPs included in the analysis increased. The highest predictive ability for DM was 0.34 (137,191 SNPs having average depth higher than 10), for HD was 0.77 (185,297 SNPs having average depth lower than 60), and for RUST was 0.55 (188,832 SNPs having average depth higher than 1).

### Heritabilities and variance components

Several studies have reported heritabilities in diploid ryegrass for the same traits studied here. Fè et al. (2015b) reported analysis of total DM in two years, and heritability ranged from 0.20 to 0.25, and the estimates of heritability of total DM over two years were slightly higher than in first and second year separately. In the current study, DM was defined as the total dry matter yield in each of two farming years, and modeled as a trait with repeated records while the overall year effect was included in the fixed effects. The estimate of $h_{f}^{2}$ was higher than heritabilities reported by Fè et al. (2015b). Compared with the heritability for HD in diploids, where estimates ranged from 0.49 to 0.68 (Fè et al., 2015a,b) and from 0.07 to 0.22 (Ashraf et al., 2016), the estimates in the current study are higher. RUST was investigated in diploid varieties by Ravel and Charmet (1996), Waldron et al. (1998), Fè et al. (2015b), and Fè et al. (2016), and the estimates of $h_{f}^{2}$ in the current study were in the range reported for diploid ryegrass. In the previous study on diploid ryegrass by Fè et al. (2015b), estimated heritability for DM was similar to that for RUST, but in the current study we find a larger difference between estimated heritabilities for DM and RUST.

G×E effects accounted for about 10% of total variance for HD in diploid ryegrass (Fè et al., 2015a), which is similar to results in the current study. Although the proportion of total phenotypic variance explained by genetic marker information was much less in DM than in HD and RUST, the variances of G×E effects were also small in DM.

The proportions of variances due to the two G×E effects were different for DM and RUST. For DM, the second G×E effect (GSLMF) was important, but the first G×E effect (GSLM) explained only a small part, which indicated that growth season had a large effect on DM and can modify the ranking of different families. In diploid ryegrass, the genetic and phenotypic correlation between DM in the two years were 0.62 and 0.39 respectively (Fè et al., 2015b), which also implies large G×E effects. Variance of G×E effects in RUST was different from G×E in DM. For RUST, both the first and the second G×E effects accounted for similar amount of variances (around 12% of total phenotypic variance), which is comparable to results from the previous study on diploids (Fè et al., 2016).

The proportion of residual variance at the level of single plots was different among the three traits. The residual variance accounted for more than 50% of phenotypic variance in DM but only around 25% in both HD and RUST. The large amount of residual variance in DM indicates larger measurement errors in DM, and necessarily leaves only relatively small proportions of variance that can be attributed to the other effects.

### SNP filtering strategies

Sequencing depth is an important factor when utilizing GBS data. An increase of sequencing depth means that the average number of times a locus been sequenced is increased, so that the accuracy of measuring the frequency of the reference allele is also increased. However, increasing sequencing depth also increases the cost of sequencing. Therefore, it is crucial to investigate the optimal sequencing depth when using GBS data. In the current study, different SNP filtering scenarios were compared with regard to parameter estimation and genomic prediction results. Four SNP filtering strategies were applied on the full GBS dataset, creating subsets of SNPs with different sequencing depth and/or different numbers of SNPs.

A previous study on diploid ryegrass (Ashraf et al., 2016) used GBS data with sequencing depth varying from 0 to 60, and divided the SNPs in 5 groups with depth interval of 10. Ashraf et al. (2016) did not correct for low accuracy of allele frequency estimates at low sequencing depth, and showed this creates a general trend of increasing genomic heritability with increasing sequencing depth. In the current study, we corrected for the effects of low accuracy at low sequencing depth, based on Cericola et al. (2018), and generally see no more clear linear correlation between sequencing depth and heritability. For instance, the FILTBOTH strategy also grouped SNPs into different depth intervals, and highest estimates of genomic heritability were found for the middle to lower levels FILTBOTH2 (depth 5–10) and FILTBOTH3 (depth 10–20). Comparable heritabilities were found between FILTBOTH 3 and RAN80, where both scenarios included similar amount of SNPs while the later one covering a larger range of sequencing depths (1–278). Hence, the corrections for bias from Cericola et al. (2018) are removing obvious trends related to sequencing depth, and seem to effectively remedy the problem of biased heritabilities at low sequencing depth reported by Ashraf et al. (2016).

For prediction accuracy, the impact from GBS sequencing depth was investigated in simulated biparental segregating populations (Gorjanc et al., 2017) as well as in outbred livestock populations (Gorjanc et al., 2015). The results from these two simulation studies showed that GBS data with low coverage (~1X) could provide prediction accuracy comparable to SNP array data. When using field data, most of the studies were focused on settings with inbred individuals, e.g., in wheat (Poland et al., 2012) and maize (Crossa et al., 2013). The accuracy of genomic prediction using low-coverage GBS data were comparable with SNP array or diversity array technology data in inbred populations (Poland et al., 2012; Crossa et al., 2013). Different from these simulation studies or studies on inbred populations, the current study is based on the commercial tetraploid data using family-pools. In our data, we cannot confirm that GBS data with low sequencing depth of about 1X already gives accurate predictions. As expected, high heterozygosity in tetraploid ryegrass, combined with use of family-pools, requires higher sequencing depth for accurate genomic prediction. In the current study, SNPs with sequencing depth between 10 and 20 (FILTBOTH3) delivered desirable predictive ability.

In the current study, by filtering out SNPs with either low sequencing depth (FILTLOW) or with high sequencing depth (FILTHIGH), the optimal sequencing depth for practical genomic prediction in tetraploid ryegrass was investigated. In FILTLOW groups, FILTLOW1 to FILTLOW3 gave most accurate predictions. The number of SNPs included in the models with highest predictive ability was about 140–180 k. The lowest sequencing depth for SNPs in FILTLOW3 was 10. The similar high predictive ability provided by FILTLOW1 to FILTLOW3 indicated that excluding low sequencing depth (1–10) SNPs did not affect the predictive ability significantly. In FILTHIGH groups, FILTHIGH1 to FILTHIGH9 gave similar predictive abilities, which indicated that accurate predictions can be reached even by including only SNPs with sequencing depth lower than 20. This can simply be an effect of still having sufficiently large numbers of SNPs with depth lower than 20, and removing SNPs with high depth may reduce some noise caused by repetitive sequences. Hence, filtering out SNPs with high depth can increase the proportion of useful information without reducing the prediction accuracy. Compared with the RAN strategy, filtering out SNPs with low depth provided higher predictive ability than using a similar number of randomly chosen SNPs, and when comparing the FILTHIGH strategy with the RAN strategy, filtering out SNPs with high depth provided similar predictive ability as using a similar number of randomly chosen SNPs. For the three traits investigated in the current study, the best predictive abilities were not achieved with exactly the same filtering strategy, however, differences between the best filtering strategies were small. In practical breeding, single trait evaluation can be carried out by using **G** matrices built from different sets of SNPs. It is also feasible to apply index selection on a combination of traits with different weights by using a same set of SNPs, which can provide globally accurate predictions. For example, in the current study, even though the highest predictive ability was provided by FILTLOW3, FILTHIGH5, and FILTLOW1 for DM, HD, and RUST, respectively, FILTLOW3 can be chosen as a scenario that provided accurate predictions for all the three traits analyzed. In addition, applying different sets of SNPs at the same time is also achievable by using random regression models disregarding the higher demand of computing resources.

In addition to genomic prediction accuracy, bias was also investigated in this study. In general, it was observed that predictions were biased, and with increasing number of SNPs included in the model, more biased predictions were observed. This can be due to many factors. The definition of the **G** matrix could be one of the reasons. When using GBS data, the allele frequencies can suffer from inaccuracy due to low sequencing depth, which can induce bias into the prediction. However, in the current study, biases due to low sequencing depth was corrected for using the method reported by Cericola et al. (2018). In addition, G×E interactions were modeled in a rather simple way, and bias of prediction may be reduced by better modeling of G×E effects (Fè et al., 2015b).

For diploid heterozygotic species, a minimum sequencing depth of around 10X is needed to obtain accurate calling (Chenuil, 2012). However, for tetraploid species, the requirement of sequencing depth for accurate calling of tetraploid genotypes was reported to be 60–80X (Uitdewilligen et al., 2013). For genomic prediction, however, it is not necessary to obtain accurate calling for each individual sample. The results in the current study indicate that high predictive ability can be obtained using much lower sequencing depth because only the frequency and not the individual genotypes needs to be called.

## Conclusions

In the current study, phenotypic records for three traits dry matter yield (DM), rust resistance (RUST), and heading date (HD), as well as GBS data were used to obtain genomic predictions for 1,515 tetraploid F_2_ ryegrass families. Different SNP filtering strategies by filtering out SNPs according to average depth and number of SNPs were compared. The estimates of genomic heritability of family means were 0.45, 0.74, and 0.73 for DM, HD and RUST, respectively. The predictive ability for DM was 0.34, for HD was 0.77, and for RUST was 0.55. The estimation of genomic heritability and the predictive ability for DM, HD and RUST clearly showed that genomic prediction can be implemented in tetraploid perennial ryegrass. Comparison of different filtering strategies showed that using only SNPs with sequencing depth between 10 and 20 would not reduce predictive ability, and showed the potential to obtain accurate prediction from medium-low coverage GBS in tetraploids. Adding SNPs with sequencing depth lower than 10 in the model also lead to accurate predictions. The predictive ability generally increased as the number of SNPs included in the analysis increased. GBS data including 80–100 K SNPs were needed for accurate prediction of additive breeding values in tetraploid ryegrass. The results clearly illustrate that genomic prediction using GBS data can help to optimize the breeding program for tetraploid ryegrass.

## Author contributions

XG implemented and carried out the statistical analysis, interpreted the results and had a major role in drafting the manuscript. FC and DF contributed to the statistical analysis and the result interpretation, reviewed the manuscript. MP managed the production of the plant material and the acquisition of the phenotypic data. IL carried out part of the GBS sequencing, and subsequent bioinformatics analysis. CJ conceived the experiment and reviewed the manuscript. JJ conceived the experiment, contributed in developing the statistical models and interpreting the results, reviewed the manuscript. LJ contributed in developing the statistical models and interpreting the results, reviewed the manuscript. All authors read and approved the final manuscript.

### Conflict of interest statement

The authors declare that the research was conducted in the absence of any commercial or financial relationships that could be construed as a potential conflict of interest.

## Abbreviations

CV: cross-validation
DM: dry matter yield
FILTBOTH: the strategy filtering out SNPs having both low average and high average depth
FILTHIGH: the strategy filtering out SNPs having high average depth
FILTLOW: the strategy filtering out SNPs having low average depth
GBS: genotyping-by-sequencing
GEBVs: genomic breeding values
GP: genomic prediction
GSLM: family × sowing year × location × management effects
GSLMF: family × sowing year × location × management × farming year effects
G×E: genotype by environment
HD: heading date
LD: linkage disequilibrium
QTLs: quantitative trait loci
RAN: the strategy keeping SNPs randomly with different data sizes
RUST: rust resistance
SNP: single-nucleotide polymorphism.
