# Supplementary material for: Genomic Prediction in Tetraploid Ryegrass Using Allele Frequencies Based on Genotyping by Sequencing
Source: Front Plant Sci. 2018 Aug 15;9:1165. doi: 10.3389/fpls.2018.01165 (PMC6104567; doi:10.3389/fpls.2018.01165)
Supplement: Supplementary file 1 [file Table_1.docx]

**Sup. Table 1. Summary of different scenarios^1^ and sequencing depth filtering criteria.**

| Scenario | Lower threshold | Upper threshold | No. SNPs |
| --- | --- | --- | --- |
| FILTLOW1 | 1 |  | 188,832 |
| FILTLOW2 | 5 |  | 182,448 |
| FILTLOW3 | 10 |  | 137,191 |
| FILTLOW4 | 20 |  | 56,907 |
| FILTLOW5 | 30 |  | 24,168 |
| FILTLOW6 | 40 |  | 11,521 |
| FILTLOW7 | 50 |  | 6,230 |
| FILTLOW8 | 60 |  | 3,535 |
| FILTLOW9 | 70 |  | 2,415 |
| FILTLOW10 | 80 |  | 1,938 |
| FILTLOW11 | 90 |  | 1,587 |
| FILTHIGH1 |  | 100 | 187,516 |
| FILTHIGH2 |  | 90 | 187,245 |
| FILTHIGH3 |  | 80 | 186,894 |
| FILTHIGH4 |  | 70 | 186,417 |
| FILTHIGH5 |  | 60 | 185,297 |
| FILTHIGH6 |  | 50 | 182,602 |
| FILTHIGH7 |  | 40 | 177,312 |
| FILTHIGH8 |  | 30 | 164,667 |
| FILTHIGH9 |  | 20 | 131,931 |
| FILTHIGH10 |  | 10 | 51,650 |
| FILTHIGH11 |  | 5 | 6,389 |
| FILTBOTH1 | 1 | 5 | 6,384 |
| FILTBOTH2 | 5 | 10 | 45,257 |
| FILTBOTH3 | 10 | 20 | 80,284 |
| FILTBOTH4 | 20 | 30 | 32,739 |
| FILTBOTH5 | 30 | 40 | 12,647 |
| FILTBOTH6 | 40 | 50 | 5,291 |
| FILTBOTH7 | 50 | 60 | 2,695 |
| FILTBOTH8 | 60 | 70 | 1,120 |
| FILTBOTH9 | 70 | 80 | 477 |
| FILTBOTH10 | 80 | 90 | 351 |
| FILTBOTH11 | 90 | 100 | 271 |
| FILTBOTH12 | 100 |  | 1,316 |
| RAN5 |  |  | 5,000 |
| RAN10 |  |  | 10,000 |
| RAN20 |  |  | 20,000 |
| RAN40 |  |  | 40,000 |
| RAN60 |  |  | 60,000 |
| RAN80 |  |  | 80,000 |
| RAN100 |  |  | 100,000 |
| RAN120 |  |  | 120,000 |
| RAN140 |  |  | 140,000 |
| RAN160 |  |  | 160,000 |
| RAN180 |  |  | 180,000 |

^1^ FILTLOW = strategy filtering out SNPs having low average depth; FILTHIGH = strategy filtering out SNPs having high average depth; FILTBOTH = strategy filtering out SNPs having both low average and high average depth; RAN = strategy keeping SNPs randomly with different data size.

= strategy keeping SNPs randomly with different data size.
