# Supplementary material for: Genomic Prediction in Tetraploid Ryegrass Using Allele Frequencies Based on Genotyping by Sequencing
Source: Front Plant Sci. 2018 Aug 15;9:1165. doi: 10.3389/fpls.2018.01165 (PMC6104567; doi:10.3389/fpls.2018.01165)
Supplement: Supplementary file 2 [file Table_2.docx]

**Sup. Table 2. Estimated variance components^1^ (line 1) and their standard errors (line 2) for dry matter yield.**

| Scenario^2^ | $\bar{G^{*}}$ | $\sigma_{g}^{2}$ | $\bar{G^{*}}\sigma_{g}^{2}$ | $\sigma_{a}^{2}$ | $\sigma_{p}^{2}$ | $\sigma_{i_{1}}^{2}$ | $\sigma_{i_{2}}^{2}$ | $\sigma_{e}^{2}$ | $\sigma_{P_{f}}^{2}$ | $\sigma_{P_{p}}^{2}$ | $h_{f}^{2}$ |
| --- | --- | --- | --- | --- | --- | --- | --- | --- | --- | --- | --- |
| FILTLOW1 | 2.36 | 5.97E-04  1.22E-04 | 1.41E-03  2.87E-04 | 1.80E-04  2.47E-04 | 1.43E-03  2.92E-04 | 6.77E-09  2.88E-04 | 4.07E-04  2.93E-04 | 4.76E-03  3.21E-04 | 3.26E-03  2.53E-04 | 8.19E-03  1.97E-04 | 4.32E-01  8.28E-02 |
| FILTLOW2 | 2.40 | 5.80E-04  1.20E-04 | 1.39E-03  2.88E-04 | 1.92E-04  2.49E-04 | 1.43E-03  2.92E-04 | 1.98E-07  2.88E-04 | 4.06E-04  2.93E-04 | 4.76E-03  3.21E-04 | 3.26E-03  2.53E-04 | 8.19E-03  1.97E-04 | 4.28E-01  8.33E-02 |
| FILTLOW3 | 2.58 | 5.68E-04  1.16E-04 | 1.47E-03  2.98E-04 | 1.31E-04  2.55E-04 | 1.43E-03  2.91E-04 | 1.77E-07  2.87E-04 | 4.07E-04  2.93E-04 | 4.76E-03  3.21E-04 | 3.27E-03  2.53E-04 | 8.19E-03  1.98E-04 | 4.49E-01  8.57E-02 |
| FILTLOW4 | 2.68 | 4.92E-04  1.07E-04 | 1.32E-03  2.86E-04 | 2.62E-04  2.50E-04 | 1.43E-03  2.92E-04 | 1.61E-07  2.88E-04 | 4.11E-04  2.94E-04 | 4.77E-03  3.21E-04 | 3.25E-03  2.53E-04 | 8.19E-03  1.97E-04 | 4.05E-01  8.31E-02 |
| FILTLOW5 | 2.58 | 4.16E-04  9.72E-05 | 1.07E-03  2.50E-04 | 5.01E-04  2.30E-04 | 1.43E-03  2.92E-04 | 6.87E-10  2.88E-04 | 4.13E-04  2.94E-04 | 4.77E-03  3.21E-04 | 3.24E-03  2.52E-04 | 8.18E-03  1.96E-04 | 3.31E-01  7.36E-02 |
| FILTLOW6 | 2.41 | 3.56E-04  8.86E-05 | 8.58E-04  2.14E-04 | 6.95E-04  2.10E-04 | 1.44E-03  2.93E-04 | 7.64E-08  2.89E-04 | 4.15E-04  2.94E-04 | 4.76E-03  3.21E-04 | 3.23E-03  2.51E-04 | 8.17E-03  1.95E-04 | 2.66E-01  6.36E-02 |
| FILTLOW7 | 2.24 | 2.31E-04  8.09E-05 | 5.18E-04  1.81E-04 | 1.01E-03  2.03E-04 | 1.44E-03  2.94E-04 | 1.22E-07  2.91E-04 | 4.09E-04  2.94E-04 | 4.77E-03  3.22E-04 | 3.21E-03  2.50E-04 | 8.16E-03  1.93E-04 | 1.62E-01  5.52E-02 |
| FILTLOW8 | 1.97 | 2.14E-04  8.05E-05 | 4.22E-04  1.59E-04 | 1.10E-03  1.93E-04 | 1.44E-03  2.94E-04 | 4.91E-08  2.91E-04 | 4.09E-04  2.94E-04 | 4.78E-03  3.22E-04 | 3.20E-03  2.49E-04 | 8.15E-03  1.92E-04 | 1.32E-01  4.86E-02 |
| FILTLOW9 | 1.69 | 2.09E-04  8.35E-05 | 3.53E-04  1.41E-04 | 1.16E-03  1.87E-04 | 1.44E-03  2.95E-04 | 2.76E-07  2.92E-04 | 3.97E-04  2.95E-04 | 4.79E-03  3.23E-04 | 3.19E-03  2.49E-04 | 8.14E-03  1.91E-04 | 1.11E-01  4.35E-02 |
| FILTLOW10 | 1.54 | 2.04E-04  8.86E-05 | 3.14E-04  1.36E-04 | 1.19E-03  1.87E-04 | 1.45E-03  2.95E-04 | 9.40E-08  2.92E-04 | 3.99E-04  2.95E-04 | 4.79E-03  3.23E-04 | 3.18E-03  2.49E-04 | 8.14E-03  1.91E-04 | 9.87E-02  4.22E-02 |
| FILTLOW11 | 1.43 | 2.31E-04  9.39E-05 | 3.31E-04  1.35E-04 | 1.17E-03  1.84E-04 | 1.44E-03  2.95E-04 | 1.56E-07  2.92E-04 | 3.95E-04  2.95E-04 | 4.79E-03  3.23E-04 | 3.18E-03  2.49E-04 | 8.14E-03  1.91E-04 | 1.04E-01  4.15E-02 |
| FILTHIGH1 | 2.36 | 5.95E-04  1.21E-04 | 1.40E-03  2.86E-04 | 1.82E-04  2.47E-04 | 1.43E-03  2.92E-04 | 3.95E-08  2.88E-04 | 4.07E-04  2.93E-04 | 4.76E-03  3.21E-04 | 3.26E-03  2.53E-04 | 8.19E-03  1.97E-04 | 4.31E-01  8.26E-02 |
| FILTHIGH2 | 2.36 | 5.94E-04  1.21E-04 | 1.40E-03  2.86E-04 | 1.84E-04  2.47E-04 | 1.43E-03  2.92E-04 | 7.46E-08  2.88E-04 | 4.07E-04  2.93E-04 | 4.76E-03  3.21E-04 | 3.26E-03  2.53E-04 | 8.19E-03  1.97E-04 | 4.30E-01  8.26E-02 |
| FILTHIGH3 | 2.36 | 5.94E-04  1.21E-04 | 1.40E-03  2.86E-04 | 1.85E-04  2.46E-04 | 1.43E-03  2.92E-04 | 7.45E-08  2.88E-04 | 4.07E-04  2.93E-04 | 4.76E-03  3.21E-04 | 3.26E-03  2.53E-04 | 8.19E-03  1.97E-04 | 4.30E-01  8.25E-02 |
| FILTHIGH4 | 2.36 | 5.92E-04  1.21E-04 | 1.40E-03  2.85E-04 | 1.87E-04  2.46E-04 | 1.43E-03  2.92E-04 | 1.74E-07  2.88E-04 | 4.07E-04  2.93E-04 | 4.76E-03  3.21E-04 | 3.26E-03  2.53E-04 | 8.19E-03  1.97E-04 | 4.30E-01  8.24E-02 |
| FILTHIGH5 | 2.36 | 5.92E-04  1.21E-04 | 1.40E-03  2.85E-04 | 1.88E-04  2.46E-04 | 1.43E-03  2.92E-04 | 1.86E-07  2.88E-04 | 4.07E-04  2.93E-04 | 4.76E-03  3.21E-04 | 3.25E-03  2.53E-04 | 8.19E-03  1.97E-04 | 4.29E-01  8.23E-02 |
| FILTHIGH6 | 2.36 | 5.94E-04  1.20E-04 | 1.40E-03  2.83E-04 | 1.85E-04  2.44E-04 | 1.43E-03  2.92E-04 | 4.21E-07  2.88E-04 | 4.06E-04  2.93E-04 | 4.76E-03  3.21E-04 | 3.25E-03  2.53E-04 | 8.18E-03  1.97E-04 | 4.30E-01  8.18E-02 |
| FILTHIGH7 | 2.35 | 5.88E-04  1.20E-04 | 1.38E-03  2.81E-04 | 2.03E-04  2.43E-04 | 1.43E-03  2.92E-04 | 1.96E-07  2.88E-04 | 4.06E-04  2.93E-04 | 4.77E-03  3.21E-04 | 3.25E-03  2.53E-04 | 8.19E-03  1.97E-04 | 4.25E-01  8.13E-02 |
| FILTHIGH8 | 2.32 | 5.67E-04  1.17E-04 | 1.32E-03  2.72E-04 | 2.60E-04  2.38E-04 | 1.43E-03  2.92E-04 | 2.19E-08  2.88E-04 | 4.05E-04  2.93E-04 | 4.77E-03  3.21E-04 | 3.25E-03  2.52E-04 | 8.18E-03  1.97E-04 | 4.05E-01  7.90E-02 |
| FILTHIGH9 | 2.23 | 5.45E-04  1.13E-04 | 1.21E-03  2.51E-04 | 3.57E-04  2.25E-04 | 1.43E-03  2.92E-04 | 2.13E-07  2.88E-04 | 4.04E-04  2.93E-04 | 4.77E-03  3.21E-04 | 3.24E-03  2.52E-04 | 8.17E-03  1.96E-04 | 3.75E-01  7.34E-02 |
| FILTHIGH10 | 1.90 | 3.97E-04  9.61E-05 | 7.53E-04  1.82E-04 | 7.40E-04  1.98E-04 | 1.44E-03  2.93E-04 | 3.10E-07  2.90E-04 | 4.08E-04  2.94E-04 | 4.77E-03  3.22E-04 | 3.17E-03  2.48E-04 | 8.11E-03  1.90E-04 | 2.38E-01  5.62E-02 |
| FILTHIGH11 | 1.51 | 1.59E-04  5.19E-05 | 2.40E-04  7.85E-05 | 1.07E-03  1.83E-04 | 1.45E-03  2.95E-04 | 1.63E-08  2.91E-04 | 4.11E-04  2.95E-04 | 4.78E-03  3.22E-04 | 2.99E-03  2.44E-04 | 7.95E-03  1.85E-04 | 8.03E-02  2.73E-02 |
| FILTBOTH1 | 1.51 | 1.58E-04  5.18E-05 | 2.39E-04  7.84E-05 | 1.07E-03  1.84E-04 | 1.45E-03  2.95E-04 | 1.59E-08  2.92E-04 | 4.11E-04  2.95E-04 | 4.78E-03  3.22E-04 | 2.99E-03  2.44E-04 | 7.95E-03  1.85E-04 | 7.98E-02  2.73E-02 |
| FILTBOTH2 | 1.97 | 3.55E-04  9.16E-05 | 7.00E-04  1.81E-04 | 7.93E-04  2.00E-04 | 1.44E-03  2.94E-04 | 3.61E-07  2.90E-04 | 4.04E-04  2.94E-04 | 4.78E-03  3.22E-04 | 3.17E-03  2.48E-04 | 8.11E-03  1.90E-04 | 2.21E-01  5.59E-02 |
| FILTBOTH3 | 2.51 | 4.87E-04  1.01E-04 | 1.22E-03  2.53E-04 | 3.61E-04  2.25E-04 | 1.43E-03  2.92E-04 | 3.16E-07  2.88E-04 | 4.04E-04  2.93E-04 | 4.77E-03  3.21E-04 | 3.25E-03  2.52E-04 | 8.18E-03  1.97E-04 | 3.75E-01  7.36E-02 |
| FILTBOTH4 | 2.75 | 3.72E-04  8.79E-05 | 1.02E-03  2.41E-04 | 5.24E-04  2.26E-04 | 1.44E-03  2.93E-04 | 1.09E-07  2.90E-04 | 4.06E-04  2.94E-04 | 4.78E-03  3.22E-04 | 3.22E-03  2.51E-04 | 8.17E-03  1.95E-04 | 3.17E-01  7.16E-02 |
| FILTBOTH5 | 2.71 | 3.04E-04  7.90E-05 | 8.23E-04  2.14E-04 | 7.31E-04  2.11E-04 | 1.43E-03  2.93E-04 | 6.81E-08  2.89E-04 | 4.08E-04  2.95E-04 | 4.78E-03  3.22E-04 | 3.23E-03  2.51E-04 | 8.17E-03  1.95E-04 | 2.55E-01  6.38E-02 |
| FILTBOTH6 | 2.57 | 2.60E-04  6.52E-05 | 6.68E-04  1.68E-04 | 8.39E-04  1.88E-04 | 1.44E-03  2.93E-04 | 9.21E-08  2.90E-04 | 4.14E-04  2.94E-04 | 4.77E-03  3.22E-04 | 3.18E-03  2.49E-04 | 8.13E-03  1.92E-04 | 2.10E-01  5.12E-02 |
| FILTBOTH7 | 2.49 | 1.19E-04  5.39E-05 | 2.95E-04  1.34E-04 | 1.19E-03  1.90E-04 | 1.45E-03  2.95E-04 | 1.38E-07  2.92E-04 | 4.06E-04  2.95E-04 | 4.78E-03  3.23E-04 | 3.17E-03  2.48E-04 | 8.12E-03  1.89E-04 | 9.33E-02  4.20E-02 |
| FILTBOTH8 | 2.37 | 5.96E-05  4.02E-05 | 1.41E-04  9.54E-05 | 1.33E-03  1.80E-04 | 1.45E-03  2.95E-04 | 2.43E-08  2.92E-04 | 4.09E-04  2.95E-04 | 4.78E-03  3.23E-04 | 3.16E-03  2.47E-04 | 8.11E-03  1.88E-04 | 4.48E-02  3.02E-02 |
| FILTBOTH9 | 2.09 | 3.79E-05  3.05E-05 | 7.93E-05  6.38E-05 | 1.39E-03  1.72E-04 | 1.44E-03  2.96E-04 | 1.19E-07  2.92E-04 | 4.00E-04  2.95E-04 | 4.80E-03  3.24E-04 | 3.15E-03  2.47E-04 | 8.11E-03  1.88E-04 | 2.52E-02  2.03E-02 |
| FILTBOTH10 | 1.86 | 2.14E-05  3.07E-05 | 3.98E-05  5.70E-05 | 1.43E-03  1.73E-04 | 1.45E-03  2.96E-04 | 4.81E-08  2.93E-04 | 4.03E-04  2.95E-04 | 4.79E-03  3.24E-04 | 3.15E-03  2.47E-04 | 8.11E-03  1.88E-04 | 1.26E-02  1.81E-02 |
| FILTBOTH11 | 1.68 | 4.72E-05  4.05E-05 | 7.91E-05  6.79E-05 | 1.38E-03  1.73E-04 | 1.45E-03  2.96E-04 | 1.05E-07  2.93E-04 | 3.99E-04  2.95E-04 | 4.80E-03  3.24E-04 | 3.15E-03  2.47E-04 | 8.11E-03  1.88E-04 | 2.51E-02  2.16E-02 |
| FILTBOTH12 | 1.36 | 1.60E-04  8.01E-05 | 2.18E-04  1.09E-04 | 1.27E-03  1.78E-04 | 1.44E-03  2.95E-04 | 2.31E-07  2.92E-04 | 3.97E-04  2.95E-04 | 4.79E-03  3.23E-04 | 3.17E-03  2.48E-04 | 8.12E-03  1.90E-04 | 6.87E-02  3.40E-02 |
| RAN5 | 2.36 | 1.51E-04  5.26E-05 | 3.56E-04  1.24E-04 | 1.07E-03  1.90E-04 | 1.44E-03  2.95E-04 | 9.68E-08  2.91E-04 | 4.05E-04  2.95E-04 | 4.78E-03  3.23E-04 | 3.11E-03  2.45E-04 | 8.06E-03  1.86E-04 | 1.15E-01  4.02E-02 |
| RAN10 | 2.36 | 2.36E-04  6.70E-05 | 5.56E-04  1.58E-04 | 9.11E-04  1.94E-04 | 1.44E-03  2.94E-04 | 1.97E-07  2.90E-04 | 4.08E-04  2.94E-04 | 4.78E-03  3.22E-04 | 3.14E-03  2.46E-04 | 8.09E-03  1.88E-04 | 1.77E-01  4.97E-02 |
| RAN20 | 2.36 | 3.34E-04  8.18E-05 | 7.87E-04  1.93E-04 | 7.33E-04  2.01E-04 | 1.43E-03  2.93E-04 | 2.06E-07  2.90E-04 | 4.05E-04  2.94E-04 | 4.78E-03  3.22E-04 | 3.19E-03  2.49E-04 | 8.14E-03  1.92E-04 | 2.46E-01  5.86E-02 |
| RAN40 | 2.36 | 4.26E-04  9.41E-05 | 1.00E-03  2.22E-04 | 5.50E-04  2.10E-04 | 1.43E-03  2.93E-04 | 1.82E-07  2.89E-04 | 4.05E-04  2.94E-04 | 4.77E-03  3.22E-04 | 3.23E-03  2.51E-04 | 8.16E-03  1.95E-04 | 3.11E-01  6.56E-02 |
| RAN60 | 2.36 | 4.97E-04  1.03E-04 | 1.17E-03  2.43E-04 | 4.01E-04  2.19E-04 | 1.43E-03  2.92E-04 | 2.56E-07  2.88E-04 | 4.06E-04  2.94E-04 | 4.77E-03  3.21E-04 | 3.24E-03  2.52E-04 | 8.18E-03  1.96E-04 | 3.61E-01  7.11E-02 |
| RAN80 | 2.36 | 5.34E-04  1.09E-04 | 1.26E-03  2.56E-04 | 3.22E-04  2.26E-04 | 1.43E-03  2.92E-04 | 2.75E-07  2.88E-04 | 4.06E-04  2.93E-04 | 4.77E-03  3.21E-04 | 3.25E-03  2.52E-04 | 8.18E-03  1.97E-04 | 3.87E-01  7.43E-02 |
| RAN100 | 2.36 | 5.26E-04  1.12E-04 | 1.24E-03  2.63E-04 | 3.35E-04  2.34E-04 | 1.43E-03  2.92E-04 | 2.04E-07  2.88E-04 | 4.04E-04  2.93E-04 | 4.77E-03  3.21E-04 | 3.24E-03  2.52E-04 | 8.18E-03  1.96E-04 | 3.82E-01  7.68E-02 |
| RAN120 | 2.36 | 5.69E-04  1.16E-04 | 1.34E-03  2.74E-04 | 2.42E-04  2.38E-04 | 1.43E-03  2.92E-04 | 2.14E-07  2.88E-04 | 4.07E-04  2.93E-04 | 4.77E-03  3.21E-04 | 3.25E-03  2.53E-04 | 8.19E-03  1.97E-04 | 4.12E-01  7.92E-02 |
| RAN140 | 2.36 | 5.74E-04  1.18E-04 | 1.35E-03  2.78E-04 | 2.31E-04  2.41E-04 | 1.43E-03  2.92E-04 | 1.64E-07  2.88E-04 | 4.05E-04  2.93E-04 | 4.77E-03  3.21E-04 | 3.25E-03  2.53E-04 | 8.18E-03  1.97E-04 | 4.16E-01  8.03E-02 |
| RAN160 | 2.36 | 5.86E-04  1.20E-04 | 1.38E-03  2.82E-04 | 2.05E-04  2.44E-04 | 1.43E-03  2.92E-04 | 2.11E-07  2.88E-04 | 4.07E-04  2.93E-04 | 4.76E-03  3.21E-04 | 3.25E-03  2.53E-04 | 8.18E-03  1.97E-04 | 4.24E-01  8.15E-02 |
| RAN180 | 2.36 | 5.92E-04  1.21E-04 | 1.40E-03  2.85E-04 | 1.91E-04  2.47E-04 | 1.43E-03  2.92E-04 | 2.26E-07  2.88E-04 | 4.06E-04  2.93E-04 | 4.76E-03  3.21E-04 | 3.26E-03  2.53E-04 | 8.19E-03  1.97E-04 | 4.29E-01  8.25E-02 |

^1^ $\bar{G^{*}}$ = mean diagonal of **G^*^** matrix; $\sigma_{g}^{2}$ = additive genomic variance; $\sigma_{a}^{2}$ = residual genetic variance; $\sigma_{p}^{2}$ = random plot variance; $\sigma_{i_{1}}^{2}$ = family × sowing year × location × management variance; $\sigma_{i_{2}}^{2}$ = family × sowing year × location × management × farming year variance; $\sigma_{e}^{2}$ = residual environment variance; $\sigma_{P_{f}}^{2}$ = phenotypic variance on individual family level; $\sigma_{P_{p}}^{2}$ = phenotypic variance on plot level; $h_{f}^{2}$ = family heritability based on multiple plots.

^2^ FILTLOW = strategy filtering out SNPs having low average depth; FILTHIGH = strategy filtering out SNPs having high average depth; FILTBOTH = strategy filtering out SNPs having both low average and high average depth; RAN = strategy keeping SNPs randomly with different data size.
