# Supplementary material for: Genomic Prediction in Tetraploid Ryegrass Using Allele Frequencies Based on Genotyping by Sequencing
Source: Front Plant Sci. 2018 Aug 15;9:1165. doi: 10.3389/fpls.2018.01165 (PMC6104567; doi:10.3389/fpls.2018.01165)
Supplement: Supplementary file 3 [file Table_3.docx]

**Sup. Table 3. Estimated variance components^1^ (line 1) and their standard errors (line 2) for heading date.**

| Scenario^2^ | $\bar{G^{*}}$ | $\sigma_{g}^{2}$ | $\bar{G^{*}}\sigma_{g}^{2}$ | $\sigma_{a}^{2}$ | $\sigma_{i_{1}}^{2}$ | $\sigma_{e}^{2}$ | $\sigma_{P_{f}}^{2}$ | $\sigma_{P_{p}}^{2}$ | $h_{f}^{2}$ |
| --- | --- | --- | --- | --- | --- | --- | --- | --- | --- |
| FILTLOW1 | 2.60 | 4.02E+00  4.35E-01 | 1.05E+01  1.13E+00 | 2.35E-06  1.16E+00 | 1.81E+00  4.93E-01 | 3.88E+00  2.08E-01 | 1.41E+01  7.27E-01 | 1.61E+01  7.27E-01 | 7.40E-01  7.31E-02 |
| FILTLOW2 | 2.67 | 3.93E+00  4.30E-01 | 1.05E+01  1.14E+00 | 9.51E-07  1.17E+00 | 1.81E+00  4.93E-01 | 3.88E+00  2.08E-01 | 1.42E+01  7.29E-01 | 1.62E+01  7.28E-01 | 7.40E-01  7.41E-02 |
| FILTLOW3 | 2.89 | 3.66E+00  4.06E-01 | 1.06E+01  1.17E+00 | 3.74E-07  1.21E+00 | 1.83E+00  4.97E-01 | 3.87E+00  2.08E-01 | 1.43E+01  7.34E-01 | 1.63E+01  7.33E-01 | 7.41E-01  7.59E-02 |
| FILTLOW4 | 3.03 | 3.50E+00  3.78E-01 | 1.06E+01  1.14E+00 | 9.08E-07  1.13E+00 | 1.98E+00  5.23E-01 | 3.87E+00  2.08E-01 | 1.44E+01  7.38E-01 | 1.65E+01  7.37E-01 | 7.34E-01  6.98E-02 |
| FILTLOW5 | 2.91 | 3.67E+00  3.76E-01 | 1.07E+01  1.09E+00 | 1.02E-07  1.02E+00 | 2.27E+00  5.78E-01 | 3.89E+00  2.10E-01 | 1.48E+01  7.61E-01 | 1.68E+01  7.60E-01 | 7.22E-01  5.90E-02 |
| FILTLOW6 | 2.71 | 3.99E+00  4.07E-01 | 1.08E+01  1.10E+00 | 1.45E-07  1.02E+00 | 3.13E+00  7.47E-01 | 3.93E+00  2.12E-01 | 1.57E+01  8.35E-01 | 1.79E+01  8.33E-01 | 6.89E-01  4.99E-02 |
| FILTLOW7 | 2.50 | 4.08E+00  4.36E-01 | 1.02E+01  1.09E+00 | 9.49E-08  1.17E+00 | 4.59E+00  1.03E+00 | 3.94E+00  2.13E-01 | 1.64E+01  8.98E-01 | 1.88E+01  8.97E-01 | 6.24E-01  4.53E-02 |
| FILTLOW8 | 2.19 | 4.03E+00  5.37E-01 | 8.85E+00  1.18E+00 | 3.01E+00  1.32E+00 | 4.90E+00  1.12E+00 | 3.96E+00  2.15E-01 | 1.83E+01  1.01E+00 | 2.07E+01  1.01E+00 | 4.84E-01  4.84E-02 |
| FILTLOW9 | 1.88 | 3.64E+00  5.81E-01 | 6.83E+00  1.09E+00 | 4.85E+00  1.44E+00 | 5.36E+00  1.21E+00 | 3.97E+00  2.16E-01 | 1.85E+01  1.01E+00 | 2.10E+01  1.00E+00 | 3.69E-01  4.82E-02 |
| FILTLOW10 | 1.72 | 3.04E+00  6.11E-01 | 5.21E+00  1.05E+00 | 6.59E+00  1.53E+00 | 5.53E+00  1.25E+00 | 3.98E+00  2.17E-01 | 1.88E+01  1.01E+00 | 2.13E+01  9.99E-01 | 2.77E-01  4.90E-02 |
| FILTLOW11 | 1.58 | 3.20E+00  6.60E-01 | 5.07E+00  1.05E+00 | 6.70E+00  1.55E+00 | 5.63E+00  1.28E+00 | 3.98E+00  2.17E-01 | 1.89E+01  1.02E+00 | 2.14E+01  1.01E+00 | 2.69E-01  4.88E-02 |
| FILTHIGH1 | 2.61 | 4.01E+00  4.34E-01 | 1.05E+01  1.13E+00 | 3.63E-07  1.15E+00 | 1.80E+00  4.93E-01 | 3.88E+00  2.08E-01 | 1.41E+01  7.27E-01 | 1.61E+01  7.26E-01 | 7.40E-01  7.28E-02 |
| FILTHIGH2 | 2.61 | 4.01E+00  4.33E-01 | 1.04E+01  1.13E+00 | 2.21E-07  1.15E+00 | 1.80E+00  4.93E-01 | 3.88E+00  2.08E-01 | 1.41E+01  7.27E-01 | 1.61E+01  7.26E-01 | 7.39E-01  7.27E-02 |
| FILTHIGH3 | 2.61 | 4.00E+00  4.33E-01 | 1.04E+01  1.13E+00 | 3.41E-07  1.15E+00 | 1.80E+00  4.93E-01 | 3.88E+00  2.08E-01 | 1.41E+01  7.27E-01 | 1.61E+01  7.26E-01 | 7.39E-01  7.26E-02 |
| FILTHIGH4 | 2.61 | 4.00E+00  4.32E-01 | 1.04E+01  1.13E+00 | 5.41E-07  1.15E+00 | 1.81E+00  4.94E-01 | 3.88E+00  2.08E-01 | 1.41E+01  7.27E-01 | 1.61E+01  7.26E-01 | 7.39E-01  7.25E-02 |
| FILTHIGH5 | 2.61 | 4.00E+00  4.32E-01 | 1.04E+01  1.13E+00 | 3.29E-07  1.14E+00 | 1.80E+00  4.93E-01 | 3.88E+00  2.08E-01 | 1.41E+01  7.26E-01 | 1.61E+01  7.26E-01 | 7.39E-01  7.24E-02 |
| FILTHIGH6 | 2.60 | 4.01E+00  4.32E-01 | 1.05E+01  1.13E+00 | 2.13E-06  1.14E+00 | 1.81E+00  4.95E-01 | 3.88E+00  2.08E-01 | 1.41E+01  7.27E-01 | 1.61E+01  7.27E-01 | 7.39E-01  7.19E-02 |
| FILTHIGH7 | 2.59 | 4.03E+00  4.31E-01 | 1.05E+01  1.12E+00 | 1.23E-06  1.13E+00 | 1.84E+00  4.99E-01 | 3.88E+00  2.08E-01 | 1.42E+01  7.28E-01 | 1.62E+01  7.27E-01 | 7.38E-01  7.07E-02 |
| FILTHIGH8 | 2.56 | 4.11E+00  4.34E-01 | 1.05E+01  1.11E+00 | 9.84E-07  1.10E+00 | 1.88E+00  5.08E-01 | 3.88E+00  2.09E-01 | 1.43E+01  7.32E-01 | 1.63E+01  7.31E-01 | 7.37E-01  6.84E-02 |
| FILTHIGH9 | 2.43 | 4.33E+00  4.35E-01 | 1.05E+01  1.06E+00 | 1.06E-06  1.01E+00 | 2.01E+00  5.32E-01 | 3.90E+00  2.10E-01 | 1.44E+01  7.41E-01 | 1.64E+01  7.40E-01 | 7.31E-01  6.08E-02 |
| FILTHIGH10 | 2.02 | 4.76E+00  4.52E-01 | 9.64E+00  9.16E-01 | 2.06E-07  9.50E-01 | 3.27E+00  7.72E-01 | 3.94E+00  2.13E-01 | 1.46E+01  7.72E-01 | 1.68E+01  7.71E-01 | 6.59E-01  4.38E-02 |
| FILTHIGH11 | 1.56 | 2.94E+00  3.77E-01 | 4.58E+00  5.88E-01 | 3.95E+00  1.23E+00 | 4.62E+00  1.06E+00 | 3.95E+00  2.14E-01 | 1.47E+01  7.37E-01 | 1.71E+01  7.36E-01 | 3.12E-01  3.69E-02 |
| FILTBOTH1 | 1.56 | 2.94E+00  3.77E-01 | 4.58E+00  5.87E-01 | 3.93E+00  1.23E+00 | 4.63E+00  1.06E+00 | 3.95E+00  2.14E-01 | 1.47E+01  7.37E-01 | 1.71E+01  7.36E-01 | 3.12E-01  3.69E-02 |
| FILTBOTH2 | 2.12 | 4.60E+00  4.40E-01 | 9.75E+00  9.33E-01 | 2.24E-07  9.59E-01 | 3.27E+00  7.73E-01 | 3.94E+00  2.13E-01 | 1.48E+01  7.80E-01 | 1.70E+01  7.79E-01 | 6.61E-01  4.43E-02 |
| FILTBOTH3 | 2.78 | 3.87E+00  3.92E-01 | 1.08E+01  1.09E+00 | 4.07E-07  1.03E+00 | 2.12E+00  5.53E-01 | 3.90E+00  2.10E-01 | 1.47E+01  7.56E-01 | 1.68E+01  7.55E-01 | 7.30E-01  6.07E-02 |
| FILTBOTH4 | 3.11 | 3.43E+00  3.49E-01 | 1.07E+01  1.08E+00 | 7.64E-09  1.01E+00 | 2.57E+00  6.32E-01 | 3.90E+00  2.10E-01 | 1.50E+01  7.75E-01 | 1.71E+01  7.75E-01 | 7.10E-01  5.60E-02 |
| FILTBOTH5 | 3.07 | 3.29E+00  3.29E-01 | 1.01E+01  1.01E+00 | 3.41E-07  1.00E+00 | 3.40E+00  7.97E-01 | 3.91E+00  2.11E-01 | 1.52E+01  8.06E-01 | 1.74E+01  8.05E-01 | 6.64E-01  4.68E-02 |
| FILTBOTH6 | 2.90 | 3.46E+00  3.64E-01 | 1.00E+01  1.05E+00 | 2.62E-01  1.10E+00 | 4.37E+00  9.94E-01 | 3.95E+00  2.14E-01 | 1.62E+01  8.95E-01 | 1.86E+01  8.94E-01 | 6.17E-01  4.31E-02 |
| FILTBOTH7 | 2.80 | 3.07E+00  3.49E-01 | 8.60E+00  9.78E-01 | 1.18E+00  1.18E+00 | 4.89E+00  1.09E+00 | 3.94E+00  2.14E-01 | 1.62E+01  9.01E-01 | 1.86E+01  8.99E-01 | 5.31E-01  4.17E-02 |
| FILTBOTH8 | 2.65 | 2.29E+00  3.72E-01 | 6.06E+00  9.87E-01 | 5.63E+00  1.37E+00 | 5.09E+00  1.17E+00 | 3.96E+00  2.15E-01 | 1.83E+01  1.00E+00 | 2.07E+01  9.97E-01 | 3.31E-01  4.42E-02 |
| FILTBOTH9 | 2.31 | 1.66E+00  3.48E-01 | 3.84E+00  8.03E-01 | 7.41E+00  1.48E+00 | 5.62E+00  1.27E+00 | 3.97E+00  2.16E-01 | 1.83E+01  9.85E-01 | 2.08E+01  9.78E-01 | 2.09E-01  3.87E-02 |
| FILTBOTH10 | 2.12 | 5.56E-01  2.27E-01 | 1.18E+00  4.81E-01 | 9.94E+00  1.60E+00 | 5.92E+00  1.34E+00 | 3.98E+00  2.17E-01 | 1.85E+01  9.39E-01 | 2.10E+01  9.29E-01 | 6.38E-02  2.55E-02 |
| FILTBOTH11 | 1.89 | 4.60E-01  2.57E-01 | 8.68E-01  4.85E-01 | 1.03E+01  1.65E+00 | 5.99E+00  1.36E+00 | 3.98E+00  2.17E-01 | 1.86E+01  9.43E-01 | 2.11E+01  9.33E-01 | 4.68E-02  2.58E-02 |
| FILTBOTH12 | 1.50 | 2.98E+00  6.46E-01 | 4.46E+00  9.66E-01 | 7.30E+00  1.53E+00 | 5.56E+00  1.26E+00 | 3.97E+00  2.16E-01 | 1.88E+01  1.00E+00 | 2.13E+01  9.96E-01 | 2.37E-01  4.59E-02 |
| RAN5 | 2.61 | 2.61E+00  3.14E-01 | 6.81E+00  8.19E-01 | 2.49E+00  1.26E+00 | 4.93E+00  1.12E+00 | 3.95E+00  2.14E-01 | 1.58E+01  8.19E-01 | 1.82E+01  8.16E-01 | 4.32E-01  4.17E-02 |
| RAN10 | 2.60 | 3.26E+00  3.42E-01 | 8.50E+00  8.91E-01 | 5.51E-01  1.16E+00 | 4.62E+00  1.05E+00 | 3.95E+00  2.14E-01 | 1.52E+01  8.05E-01 | 1.76E+01  8.04E-01 | 5.58E-01  4.25E-02 |
| RAN20 | 2.60 | 3.76E+00  3.68E-01 | 9.78E+00  9.59E-01 | 1.65E-02  1.02E+00 | 3.65E+00  8.48E-01 | 3.93E+00  2.13E-01 | 1.51E+01  8.00E-01 | 1.74E+01  7.99E-01 | 6.47E-01  4.44E-02 |
| RAN40 | 2.60 | 3.99E+00  3.85E-01 | 1.04E+01  1.00E+00 | 5.26E-08  9.45E-01 | 2.60E+00  6.42E-01 | 3.91E+00  2.11E-01 | 1.48E+01  7.70E-01 | 1.69E+01  7.69E-01 | 7.03E-01  5.03E-02 |
| RAN60 | 2.60 | 4.04E+00  4.00E-01 | 1.05E+01  1.04E+00 | 1.90E-07  9.80E-01 | 2.24E+00  5.73E-01 | 3.90E+00  2.10E-01 | 1.46E+01  7.52E-01 | 1.66E+01  7.52E-01 | 7.21E-01  5.64E-02 |
| RAN80 | 2.60 | 4.04E+00  4.11E-01 | 1.05E+01  1.07E+00 | 5.27E-07  1.03E+00 | 2.05E+00  5.39E-01 | 3.89E+00  2.09E-01 | 1.44E+01  7.41E-01 | 1.65E+01  7.41E-01 | 7.29E-01  6.15E-02 |
| RAN100 | 2.60 | 4.03E+00  4.18E-01 | 1.05E+01  1.09E+00 | 6.43E-07  1.06E+00 | 1.94E+00  5.18E-01 | 3.89E+00  2.09E-01 | 1.43E+01  7.34E-01 | 1.63E+01  7.34E-01 | 7.34E-01  6.51E-02 |
| RAN120 | 2.60 | 4.03E+00  4.24E-01 | 1.05E+01  1.10E+00 | 5.32E-07  1.09E+00 | 1.91E+00  5.12E-01 | 3.88E+00  2.09E-01 | 1.43E+01  7.33E-01 | 1.63E+01  7.32E-01 | 7.36E-01  6.75E-02 |
| RAN140 | 2.60 | 4.03E+00  4.28E-01 | 1.05E+01  1.11E+00 | 7.45E-07  1.11E+00 | 1.87E+00  5.05E-01 | 3.88E+00  2.09E-01 | 1.42E+01  7.30E-01 | 1.62E+01  7.29E-01 | 7.37E-01  6.95E-02 |
| RAN160 | 2.60 | 4.02E+00  4.32E-01 | 1.05E+01  1.12E+00 | 1.10E-06  1.13E+00 | 1.84E+00  4.99E-01 | 3.88E+00  2.09E-01 | 1.42E+01  7.29E-01 | 1.62E+01  7.28E-01 | 7.38E-01  7.12E-02 |
| RAN180 | 2.60 | 4.02E+00  4.35E-01 | 1.05E+01  1.13E+00 | 8.91E-07  1.15E+00 | 1.83E+00  4.97E-01 | 3.88E+00  2.08E-01 | 1.42E+01  7.28E-01 | 1.62E+01  7.27E-01 | 7.39E-01  7.25E-02 |

^1^ $\bar{G^{*}}$ = mean diagonal of **G**^*^ matrix; $\sigma_{g}^{2}$ = additive genomic variance; $\sigma_{a}^{2}$ = residual genetic variance; $\sigma_{i_{1}}^{2}$ = family × sowing year × location × management variance; $\sigma_{i_{2}}^{2}$ = family × sowing year × location × management × farming year variance; $\sigma_{P_{p}}^{2}$ = phenotypic variance on plot level; $h_{f}^{2}$ = family heritability based on multiple plots.

^2^ FILTLOW = strategy filtering out SNPs having low average depth; FILTHIGH = strategy filtering out SNPs having high average depth; FILTBOTH = strategy filtering out SNPs having both low average and high average depth; RAN = strategy keeping SNPs randomly with different data size.
