# Supplementary material for: Genomic Prediction in Tetraploid Ryegrass Using Allele Frequencies Based on Genotyping by Sequencing
Source: Front Plant Sci. 2018 Aug 15;9:1165. doi: 10.3389/fpls.2018.01165 (PMC6104567; doi:10.3389/fpls.2018.01165)
Supplement: Supplementary file 4 [file Table_4.docx]

**Sup. Table 4. Estimated variance components^1^ (line 1) and their standard errors (line 2) for rust resistance.**

| Scenario^2^ | $\bar{G^{*}}$ | $\sigma_{g}^{2}$ | $\bar{G^{*}}\sigma_{g}^{2}$ | $\sigma_{a}^{2}$ | $\sigma_{p}^{2}$ | $\sigma_{i_{1}}^{2}$ | $\sigma_{i_{2}}^{2}$ | $\sigma_{e}^{2}$ | $\sigma_{P_{f}}^{2}$ | $\sigma_{P_{p}}^{2}$ | $h_{f}^{2}$ |
| --- | --- | --- | --- | --- | --- | --- | --- | --- | --- | --- | --- |
| FILTLOW1 | 2.35 | 3.29E-01  3.59E-02 | 7.74E-01  8.45E-02 | 8.87E-08  6.98E-02 | 2.21E-01  1.25E-02 | 2.42E-01  2.88E-02 | 2.35E-01  1.26E-02 | 5.01E-01  1.09E-02 | 1.06E+00  4.96E-02 | 1.97E+00  4.90E-02 | 7.28E-01  6.49E-02 |
| FILTLOW2 | 2.40 | 3.22E-01  3.54E-02 | 7.73E-01  8.50E-02 | 1.19E-07  7.05E-02 | 2.21E-01  1.25E-02 | 2.42E-01  2.89E-02 | 2.35E-01  1.26E-02 | 5.01E-01  1.09E-02 | 1.06E+00  4.96E-02 | 1.97E+00  4.90E-02 | 7.27E-01  6.55E-02 |
| FILTLOW3 | 2.59 | 2.95E-01  3.26E-02 | 7.65E-01  8.46E-02 | 2.25E-07  7.08E-02 | 2.20E-01  1.25E-02 | 2.45E-01  2.90E-02 | 2.35E-01  1.26E-02 | 5.01E-01  1.09E-02 | 1.06E+00  4.91E-02 | 1.97E+00  4.85E-02 | 7.24E-01  6.61E-02 |
| FILTLOW4 | 2.71 | 2.73E-01  2.98E-02 | 7.39E-01  8.07E-02 | 2.89E-02  6.49E-02 | 2.20E-01  1.25E-02 | 2.54E-01  2.95E-02 | 2.34E-01  1.26E-02 | 5.01E-01  1.09E-02 | 1.06E+00  4.99E-02 | 1.98E+00  4.94E-02 | 6.95E-01  6.07E-02 |
| FILTLOW5 | 2.60 | 2.34E-01  2.72E-02 | 6.10E-01  7.08E-02 | 1.60E-01  5.94E-02 | 2.20E-01  1.25E-02 | 2.58E-01  2.98E-02 | 2.34E-01  1.26E-02 | 5.01E-01  1.09E-02 | 1.07E+00  5.00E-02 | 1.98E+00  4.94E-02 | 5.71E-01  5.43E-02 |
| FILTLOW6 | 2.42 | 1.94E-01  2.58E-02 | 4.71E-01  6.27E-02 | 3.17E-01  5.67E-02 | 2.20E-01  1.25E-02 | 2.59E-01  2.98E-02 | 2.34E-01  1.26E-02 | 5.00E-01  1.09E-02 | 1.09E+00  5.05E-02 | 2.00E+00  4.99E-02 | 4.34E-01  4.90E-02 |
| FILTLOW7 | 2.25 | 1.60E-01  2.47E-02 | 3.60E-01  5.54E-02 | 4.30E-01  5.54E-02 | 2.20E-01  1.25E-02 | 2.64E-01  3.01E-02 | 2.34E-01  1.26E-02 | 5.00E-01  1.09E-02 | 1.09E+00  5.05E-02 | 2.01E+00  4.99E-02 | 3.30E-01  4.48E-02 |
| FILTLOW8 | 1.97 | 8.68E-02  2.19E-02 | 1.71E-01  4.32E-02 | 6.17E-01  5.72E-02 | 2.20E-01  1.25E-02 | 2.66E-01  3.02E-02 | 2.34E-01  1.26E-02 | 5.00E-01  1.09E-02 | 1.09E+00  4.90E-02 | 2.01E+00  4.83E-02 | 1.57E-01  3.79E-02 |
| FILTLOW9 | 1.69 | 4.86E-02  2.04E-02 | 8.23E-02  3.45E-02 | 7.06E-01  5.77E-02 | 2.20E-01  1.25E-02 | 2.67E-01  3.03E-02 | 2.34E-01  1.26E-02 | 5.00E-01  1.09E-02 | 1.09E+00  4.84E-02 | 2.01E+00  4.78E-02 | 7.55E-02  3.12E-02 |
| FILTLOW10 | 1.55 | 5.29E-02  2.13E-02 | 8.19E-02  3.30E-02 | 7.06E-01  5.66E-02 | 2.20E-01  1.25E-02 | 2.68E-01  3.03E-02 | 2.34E-01  1.26E-02 | 5.00E-01  1.09E-02 | 1.09E+00  4.85E-02 | 2.01E+00  4.78E-02 | 7.51E-02  2.97E-02 |
| FILTLOW11 | 1.44 | 7.16E-02  2.43E-02 | 1.03E-01  3.50E-02 | 6.86E-01  5.59E-02 | 2.20E-01  1.25E-02 | 2.67E-01  3.03E-02 | 2.34E-01  1.26E-02 | 5.00E-01  1.09E-02 | 1.09E+00  4.87E-02 | 2.01E+00  4.81E-02 | 9.46E-02  3.12E-02 |
| FILTHIGH1 | 2.36 | 3.29E-01  3.58E-02 | 7.74E-01  8.44E-02 | 3.15E-08  6.96E-02 | 2.21E-01  1.25E-02 | 2.42E-01  2.88E-02 | 2.35E-01  1.26E-02 | 5.01E-01  1.09E-02 | 1.06E+00  4.96E-02 | 1.97E+00  4.91E-02 | 7.28E-01  6.47E-02 |
| FILTHIGH2 | 2.36 | 3.28E-01  3.58E-02 | 7.74E-01  8.44E-02 | 2.67E-07  6.95E-02 | 2.21E-01  1.25E-02 | 2.42E-01  2.88E-02 | 2.35E-01  1.26E-02 | 5.01E-01  1.09E-02 | 1.06E+00  4.97E-02 | 1.97E+00  4.91E-02 | 7.28E-01  6.46E-02 |
| FILTHIGH3 | 2.36 | 3.28E-01  3.58E-02 | 7.74E-01  8.43E-02 | 3.67E-07  6.95E-02 | 2.21E-01  1.25E-02 | 2.42E-01  2.88E-02 | 2.35E-01  1.26E-02 | 5.01E-01  1.09E-02 | 1.06E+00  4.97E-02 | 1.97E+00  4.91E-02 | 7.28E-01  6.46E-02 |
| FILTHIGH4 | 2.36 | 3.28E-01  3.57E-02 | 7.73E-01  8.42E-02 | 1.43E-07  6.93E-02 | 2.21E-01  1.25E-02 | 2.42E-01  2.88E-02 | 2.35E-01  1.26E-02 | 5.01E-01  1.09E-02 | 1.06E+00  4.97E-02 | 1.97E+00  4.91E-02 | 7.28E-01  6.44E-02 |
| FILTHIGH5 | 2.36 | 3.28E-01  3.57E-02 | 7.73E-01  8.41E-02 | 3.78E-08  6.91E-02 | 2.21E-01  1.25E-02 | 2.42E-01  2.89E-02 | 2.35E-01  1.26E-02 | 5.01E-01  1.09E-02 | 1.06E+00  4.97E-02 | 1.97E+00  4.91E-02 | 7.27E-01  6.42E-02 |
| FILTHIGH6 | 2.35 | 3.29E-01  3.56E-02 | 7.74E-01  8.37E-02 | 1.97E-07  6.86E-02 | 2.21E-01  1.25E-02 | 2.43E-01  2.89E-02 | 2.35E-01  1.26E-02 | 5.01E-01  1.09E-02 | 1.06E+00  4.98E-02 | 1.97E+00  4.92E-02 | 7.27E-01  6.37E-02 |
| FILTHIGH7 | 2.35 | 3.29E-01  3.53E-02 | 7.72E-01  8.28E-02 | 2.57E-08  6.73E-02 | 2.20E-01  1.25E-02 | 2.45E-01  2.90E-02 | 2.35E-01  1.26E-02 | 5.01E-01  1.09E-02 | 1.06E+00  4.99E-02 | 1.97E+00  4.93E-02 | 7.26E-01  6.25E-02 |
| FILTHIGH8 | 2.32 | 3.32E-01  3.49E-02 | 7.70E-01  8.08E-02 | 2.21E-03  6.44E-02 | 2.20E-01  1.25E-02 | 2.51E-01  2.93E-02 | 2.35E-01  1.26E-02 | 5.01E-01  1.09E-02 | 1.07E+00  5.03E-02 | 1.98E+00  4.97E-02 | 7.22E-01  5.97E-02 |
| FILTHIGH9 | 2.21 | 3.09E-01  3.25E-02 | 6.83E-01  7.19E-02 | 8.52E-02  5.85E-02 | 2.20E-01  1.25E-02 | 2.55E-01  2.96E-02 | 2.34E-01  1.26E-02 | 5.01E-01  1.09E-02 | 1.06E+00  5.00E-02 | 1.98E+00  4.94E-02 | 6.42E-01  5.35E-02 |
| FILTHIGH10 | 1.87 | 2.25E-01  2.91E-02 | 4.22E-01  5.46E-02 | 3.53E-01  5.31E-02 | 2.20E-01  1.25E-02 | 2.55E-01  2.96E-02 | 2.34E-01  1.26E-02 | 5.01E-01  1.09E-02 | 1.07E+00  4.91E-02 | 1.98E+00  4.85E-02 | 3.94E-01  4.39E-02 |
| FILTHIGH11 | 1.49 | 6.57E-02  1.49E-02 | 9.79E-02  2.21E-02 | 6.19E-01  5.40E-02 | 2.20E-01  1.25E-02 | 2.65E-01  3.02E-02 | 2.34E-01  1.26E-02 | 5.00E-01  1.09E-02 | 1.02E+00  4.58E-02 | 1.94E+00  4.53E-02 | 9.62E-02  2.21E-02 |
| FILTBOTH1 | 1.49 | 6.56E-02  1.49E-02 | 9.75E-02  2.21E-02 | 6.19E-01  5.40E-02 | 2.20E-01  1.25E-02 | 2.65E-01  3.02E-02 | 2.34E-01  1.26E-02 | 5.00E-01  1.09E-02 | 1.02E+00  4.58E-02 | 1.94E+00  4.53E-02 | 9.58E-02  2.20E-02 |
| FILTBOTH2 | 1.95 | 2.06E-01  2.77E-02 | 4.01E-01  5.41E-02 | 3.72E-01  5.40E-02 | 2.20E-01  1.25E-02 | 2.57E-01  2.97E-02 | 2.34E-01  1.26E-02 | 5.00E-01  1.09E-02 | 1.07E+00  4.90E-02 | 1.99E+00  4.84E-02 | 3.75E-01  4.41E-02 |
| FILTBOTH3 | 2.51 | 2.55E-01  2.79E-02 | 6.39E-01  7.01E-02 | 1.24E-01  5.89E-02 | 2.20E-01  1.25E-02 | 2.58E-01  2.98E-02 | 2.34E-01  1.26E-02 | 5.01E-01  1.09E-02 | 1.06E+00  4.96E-02 | 1.98E+00  4.90E-02 | 6.03E-01  5.36E-02 |
| FILTBOTH4 | 2.78 | 2.05E-01  2.43E-02 | 5.69E-01  6.75E-02 | 2.08E-01  5.81E-02 | 2.20E-01  1.25E-02 | 2.56E-01  2.96E-02 | 2.34E-01  1.26E-02 | 5.01E-01  1.09E-02 | 1.07E+00  4.99E-02 | 1.99E+00  4.93E-02 | 5.30E-01  5.21E-02 |
| FILTBOTH5 | 2.74 | 1.75E-01  2.16E-02 | 4.82E-01  5.93E-02 | 2.94E-01  5.35E-02 | 2.20E-01  1.25E-02 | 2.60E-01  2.98E-02 | 2.34E-01  1.26E-02 | 5.00E-01  1.09E-02 | 1.07E+00  5.01E-02 | 1.99E+00  4.95E-02 | 4.48E-01  4.60E-02 |
| FILTBOTH6 | 2.59 | 1.15E-01  1.89E-02 | 3.00E-01  4.91E-02 | 4.89E-01  5.40E-02 | 2.20E-01  1.25E-02 | 2.59E-01  2.98E-02 | 2.34E-01  1.26E-02 | 5.00E-01  1.09E-02 | 1.09E+00  4.95E-02 | 2.00E+00  4.89E-02 | 2.76E-01  4.08E-02 |
| FILTBOTH7 | 2.50 | 1.06E-01  1.79E-02 | 2.65E-01  4.49E-02 | 5.15E-01  5.24E-02 | 2.20E-01  1.25E-02 | 2.65E-01  3.02E-02 | 2.34E-01  1.26E-02 | 5.00E-01  1.09E-02 | 1.08E+00  4.96E-02 | 2.00E+00  4.90E-02 | 2.45E-01  3.75E-02 |
| FILTBOTH8 | 2.38 | 4.21E-02  1.26E-02 | 1.00E-01  3.00E-02 | 6.78E-01  5.41E-02 | 2.20E-01  1.25E-02 | 2.68E-01  3.04E-02 | 2.34E-01  1.26E-02 | 5.00E-01  1.09E-02 | 1.08E+00  4.82E-02 | 2.00E+00  4.75E-02 | 9.26E-02  2.70E-02 |
| FILTBOTH9 | 2.08 | 2.96E-03  6.68E-03 | 6.14E-03  1.39E-02 | 7.79E-01  5.45E-02 | 2.20E-01  1.25E-02 | 2.70E-01  3.05E-02 | 2.34E-01  1.26E-02 | 5.00E-01  1.09E-02 | 1.09E+00  4.82E-02 | 2.01E+00  4.76E-02 | 5.64E-03  1.28E-02 |
| FILTBOTH10 | 1.87 | 2.05E-03  6.33E-03 | 3.84E-03  1.18E-02 | 7.82E-01  5.39E-02 | 2.20E-01  1.25E-02 | 2.70E-01  3.05E-02 | 2.34E-01  1.26E-02 | 5.00E-01  1.09E-02 | 1.09E+00  4.82E-02 | 2.01E+00  4.75E-02 | 3.52E-03  1.09E-02 |
| FILTBOTH11 | 1.68 | 9.69E-03  8.30E-03 | 1.63E-02  1.39E-02 | 7.69E-01  5.34E-02 | 2.20E-01  1.25E-02 | 2.69E-01  3.04E-02 | 2.34E-01  1.26E-02 | 5.00E-01  1.09E-02 | 1.09E+00  4.81E-02 | 2.01E+00  4.75E-02 | 1.50E-02  1.28E-02 |
| FILTBOTH12 | 1.37 | 6.49E-02  2.35E-02 | 8.90E-02  3.22E-02 | 6.98E-01  5.53E-02 | 2.20E-01  1.25E-02 | 2.68E-01  3.04E-02 | 2.34E-01  1.26E-02 | 5.00E-01  1.09E-02 | 1.09E+00  4.86E-02 | 2.01E+00  4.80E-02 | 8.17E-02  2.89E-02 |
| RAN5 | 2.36 | 7.58E-02  1.50E-02 | 1.79E-01  3.55E-02 | 5.73E-01  5.43E-02 | 2.20E-01  1.25E-02 | 2.64E-01  3.01E-02 | 2.34E-01  1.26E-02 | 5.00E-01  1.09E-02 | 1.05E+00  4.68E-02 | 1.97E+00  4.62E-02 | 1.70E-01  3.25E-02 |
| RAN10 | 2.35 | 1.25E-01  1.93E-02 | 2.94E-01  4.53E-02 | 4.64E-01  5.34E-02 | 2.20E-01  1.25E-02 | 2.62E-01  3.00E-02 | 2.34E-01  1.26E-02 | 5.00E-01  1.09E-02 | 1.06E+00  4.78E-02 | 1.98E+00  4.72E-02 | 2.78E-01  3.91E-02 |
| RAN20 | 2.35 | 1.66E-01  2.25E-02 | 3.91E-01  5.30E-02 | 3.77E-01  5.39E-02 | 2.20E-01  1.25E-02 | 2.59E-01  2.98E-02 | 2.34E-01  1.26E-02 | 5.00E-01  1.09E-02 | 1.07E+00  4.88E-02 | 1.98E+00  4.82E-02 | 3.67E-01  4.36E-02 |
| RAN40 | 2.35 | 2.24E-01  2.63E-02 | 5.27E-01  6.17E-02 | 2.45E-01  5.49E-02 | 2.20E-01  1.25E-02 | 2.57E-01  2.97E-02 | 2.34E-01  1.26E-02 | 5.01E-01  1.09E-02 | 1.07E+00  4.96E-02 | 1.98E+00  4.91E-02 | 4.93E-01  4.79E-02 |
| RAN60 | 2.35 | 2.52E-01  2.83E-02 | 5.93E-01  6.67E-02 | 1.77E-01  5.69E-02 | 2.20E-01  1.25E-02 | 2.56E-01  2.96E-02 | 2.34E-01  1.26E-02 | 5.01E-01  1.09E-02 | 1.07E+00  4.97E-02 | 1.98E+00  4.91E-02 | 5.56E-01  5.11E-02 |
| RAN80 | 2.35 | 2.77E-01  3.06E-02 | 6.51E-01  7.20E-02 | 1.18E-01  5.98E-02 | 2.20E-01  1.25E-02 | 2.56E-01  2.96E-02 | 2.34E-01  1.26E-02 | 5.01E-01  1.09E-02 | 1.07E+00  4.99E-02 | 1.98E+00  4.93E-02 | 6.11E-01  5.46E-02 |
| RAN100 | 2.35 | 3.00E-01  3.18E-02 | 7.05E-01  7.49E-02 | 5.86E-02  6.08E-02 | 2.20E-01  1.25E-02 | 2.56E-01  2.96E-02 | 2.34E-01  1.26E-02 | 5.01E-01  1.09E-02 | 1.06E+00  4.98E-02 | 1.97E+00  4.92E-02 | 6.65E-01  5.62E-02 |
| RAN120 | 2.35 | 3.19E-01  3.32E-02 | 7.51E-01  7.81E-02 | 1.33E-02  6.24E-02 | 2.20E-01  1.25E-02 | 2.54E-01  2.95E-02 | 2.34E-01  1.26E-02 | 5.01E-01  1.09E-02 | 1.06E+00  4.99E-02 | 1.97E+00  4.94E-02 | 7.09E-01  5.80E-02 |
| RAN140 | 2.35 | 3.25E-01  3.41E-02 | 7.64E-01  8.03E-02 | 2.26E-03  6.45E-02 | 2.20E-01  1.25E-02 | 2.51E-01  2.94E-02 | 2.34E-01  1.26E-02 | 5.01E-01  1.09E-02 | 1.06E+00  4.99E-02 | 1.97E+00  4.93E-02 | 7.20E-01  5.99E-02 |
| RAN160 | 2.35 | 3.27E-01  3.50E-02 | 7.69E-01  8.24E-02 | 1.14E-07  6.70E-02 | 2.20E-01  1.25E-02 | 2.47E-01  2.91E-02 | 2.35E-01  1.26E-02 | 5.01E-01  1.09E-02 | 1.06E+00  4.98E-02 | 1.97E+00  4.92E-02 | 7.25E-01  6.24E-02 |
| RAN180 | 2.35 | 3.29E-01  3.57E-02 | 7.73E-01  8.40E-02 | 1.08E-07  6.91E-02 | 2.21E-01  1.25E-02 | 2.43E-01  2.89E-02 | 2.35E-01  1.26E-02 | 5.01E-01  1.09E-02 | 1.06E+00  4.97E-02 | 1.97E+00  4.91E-02 | 7.27E-01  6.42E-02 |

^1^ $\bar{G^{*}}$ = mean diagonal of **G^*^** matrix; $\sigma_{g}^{2}$ = additive genomic variance; $\sigma_{a}^{2}$ = residual genetic variance; $\sigma_{p}^{2}$ = random plot variance; $\sigma_{i_{1}}^{2}$ = family × sowing year × location × management variance; $\sigma_{i_{2}}^{2}$ = family × sowing year × location × management × farming year variance; $\sigma_{e}^{2}$ = residual environment variance; $\sigma_{P_{f}}^{2}$ = phenotypic variance on individual family level; $\sigma_{P_{p}}^{2}$ = phenotypic variance on plot level; $h_{f}^{2}$ = family heritability based on multiple plots.

^2^ FILTLOW = strategy filtering out SNPs having low average depth; FILTHIGH = strategy filtering out SNPs having high average depth; FILTBOTH = strategy filtering out SNPs having both low average and high average depth; RAN = strategy keeping SNPs randomly with different data size.
