# Supplementary material for: Genomic Prediction in Tetraploid Ryegrass Using Allele Frequencies Based on Genotyping by Sequencing
Source: Front Plant Sci. 2018 Aug 15;9:1165. doi: 10.3389/fpls.2018.01165 (PMC6104567; doi:10.3389/fpls.2018.01165)
Supplement: Supplementary file 5 [file Table_5.docx]

**Sup. Table 5. Predictive ability (Cor) and bias^1^ (Reg) for three traits^2^.**

| Trait  Scenario | DM | | HD | | RUST | |
| --- | --- | --- | --- | --- | --- | --- |
|  | Cor | Reg | Cor | Reg | Cor | Reg |
| FILTLOW1 | 0.34 (0.03) | 1.30 (0.11) | 0.77 (0.02) | 1.25 (0.03) | 0.55 (0.02) | 1.27 (0.05) |
| FILTLOW2 | 0.33 (0.03) | 1.31 (0.11) | 0.77 (0.02) | 1.25 (0.03) | 0.55 (0.02) | 1.27 (0.05) |
| FILTLOW3 | 0.34 (0.03) | 1.32 (0.11) | 0.76 (0.02) | 1.26 (0.03) | 0.54 (0.02) | 1.27 (0.05) |
| FILTLOW4 | 0.32 (0.03) | 1.25 (0.11) | 0.75 (0.02) | 1.22 (0.03) | 0.50 (0.02) | 1.18 (0.05) |
| FILTLOW5 | 0.29 (0.03) | 1.25 (0.12) | 0.73 (0.02) | 1.16 (0.03) | 0.47 (0.02) | 1.15 (0.06) |
| FILTLOW6 | 0.26 (0.03) | 1.18 (0.13) | 0.69 (0.02) | 1.08 (0.04) | 0.42 (0.02) | 1.16 (0.07) |
| FILTLOW7 | 0.18 (0.03) | 1.13 (0.18) | 0.63 (0.02) | 1.03 (0.04) | 0.36 (0.02) | 1.16 (0.08) |
| FILTLOW8 | 0.18 (0.03) | 1.29 (0.20) | 0.51 (0.03) | 1.04 (0.06) | 0.23 (0.03) | 1.28 (0.14) |
| FILTLOW9 | 0.17 (0.03) | 1.25 (0.21) | 0.42 (0.03) | 1.05 (0.07) | 0.15 (0.03) | 1.46 (0.25) |
| FILTLOW10 | 0.14 (0.03) | 1.16 (0.23) | 0.33 (0.03) | 1.07 (0.10) | 0.14 (0.03) | 1.33 (0.24) |
| FILTLOW11 | 0.15 (0.03) | 1.12 (0.21) | 0.32 (0.03) | 1.06 (0.10) | 0.16 (0.03) | 1.24 (0.19) |
| FILTHIGH1 | 0.34 (0.03) | 1.30 (0.11) | 0.77 (0.02) | 1.24 (0.03) | 0.55 (0.02) | 1.27 (0.05) |
| FILTHIGH2 | 0.34 (0.03) | 1.30 (0.11) | 0.77 (0.02) | 1.24 (0.03) | 0.55 (0.02) | 1.27 (0.05) |
| FILTHIGH3 | 0.34 (0.03) | 1.30 (0.11) | 0.77 (0.02) | 1.24 (0.03) | 0.55 (0.02) | 1.27 (0.05) |
| FILTHIGH4 | 0.34 (0.03) | 1.30 (0.11) | 0.77 (0.02) | 1.24 (0.03) | 0.55 (0.02) | 1.26 (0.05) |
| FILTHIGH5 | 0.34 (0.03) | 1.29 (0.11) | 0.77 (0.02) | 1.24 (0.03) | 0.55 (0.02) | 1.26 (0.05) |
| FILTHIGH6 | 0.34 (0.03) | 1.29 (0.10) | 0.77 (0.02) | 1.24 (0.03) | 0.55 (0.02) | 1.25 (0.05) |
| FILTHIGH7 | 0.33 (0.03) | 1.29 (0.11) | 0.77 (0.02) | 1.23 (0.03) | 0.54 (0.02) | 1.24 (0.05) |
| FILTHIGH8 | 0.32 (0.03) | 1.27 (0.11) | 0.76 (0.02) | 1.23 (0.03) | 0.53 (0.02) | 1.21 (0.05) |
| FILTHIGH9 | 0.31 (0.03) | 1.25 (0.11) | 0.76 (0.02) | 1.19 (0.03) | 0.51 (0.02) | 1.19 (0.05) |
| FILTHIGH10 | 0.24 (0.03) | 1.13 (0.13) | 0.71 (0.02) | 1.08 (0.03) | 0.42 (0.02) | 1.16 (0.06) |
| FILTHIGH11 | 0.16 (0.03) | 1.05 (0.18) | 0.53 (0.03) | 1.04 (0.05) | 0.23 (0.03) | 1.14 (0.13) |
| FILTBOTH1 | 0.16 (0.03) | 1.05 (0.18) | 0.53 (0.03) | 1.04 (0.05) | 0.22 (0.03) | 1.14 (0.13) |
| FILTBOTH2 | 0.23 (0.03) | 1.14 (0.14) | 0.71 (0.02) | 1.08 (0.03) | 0.40 (0.02) | 1.17 (0.07) |
| FILTBOTH3 | 0.31 (0.03) | 1.26 (0.11) | 0.74 (0.02) | 1.19 (0.03) | 0.49 (0.02) | 1.18 (0.05) |
| FILTBOTH4 | 0.27 (0.03) | 1.15 (0.12) | 0.71 (0.02) | 1.14 (0.04) | 0.45 (0.02) | 1.15 (0.06) |
| FILTBOTH5 | 0.25 (0.03) | 1.20 (0.14) | 0.69 (0.02) | 1.06 (0.04) | 0.43 (0.02) | 1.11 (0.06) |
| FILTBOTH6 | 0.24 (0.03) | 1.13 (0.13) | 0.65 (0.02) | 1.03 (0.04) | 0.34 (0.02) | 1.14 (0.08) |
| FILTBOTH7 | 0.12 (0.03) | 0.97 (0.24) | 0.63 (0.02) | 1.03 (0.04) | 0.33 (0.02) | 1.11 (0.08) |
| FILTBOTH8 | 0.10 (0.03) | 1.34 (0.39) | 0.45 (0.03) | 1.04 (0.07) | 0.19 (0.03) | 1.22 (0.16) |
| FILTBOTH9 | 0.09 (0.03) | 1.48 (0.45) | 0.36 (0.03) | 1.05 (0.09) | 0.05 (0.03) | 2.88 (1.49) |
| FILTBOTH10 | 0.05 (0.03) | 1.43 (0.86) | 0.17 (0.03) | 1.15 (0.21) | 0.03 (0.03) | 2.56 (2.06) |
| FILTBOTH11 | 0.06 (0.03) | 1.05 (0.50) | 0.11 (0.03) | 1.06 (0.30) | 0.07 (0.03) | 1.42 (0.50) |
| FILTBOTH12 | 0.13 (0.03) | 1.14 (0.26) | 0.31 (0.03) | 1.07 (0.11) | 0.15 (0.03) | 1.19 (0.20) |
| RAN5 | 0.16 (0.03) | 1.12 (0.21) | 0.56 (0.03) | 1.04 (0.05) | 0.27 (0.02) | 1.15 (0.11) |
| RAN10 | 0.21 (0.03) | 1.14 (0.16) | 0.64 (0.02) | 1.04 (0.04) | 0.35 (0.02) | 1.15 (0.08) |
| RAN20 | 0.25 (0.03) | 1.16 (0.13) | 0.68 (0.02) | 1.06 (0.04) | 0.40 (0.02) | 1.15 (0.07) |
| RAN40 | 0.29 (0.03) | 1.21 (0.12) | 0.73 (0.02) | 1.12 (0.03) | 0.46 (0.02) | 1.17 (0.06) |
| RAN60 | 0.31 (0.03) | 1.22 (0.11) | 0.74 (0.02) | 1.16 (0.03) | 0.48 (0.02) | 1.17 (0.06) |
| RAN80 | 0.32 (0.03) | 1.24 (0.11) | 0.75 (0.02) | 1.19 (0.03) | 0.49 (0.02) | 1.18 (0.05) |
| RAN100 | 0.31 (0.03) | 1.25 (0.11) | 0.76 (0.02) | 1.21 (0.03) | 0.51 (0.02) | 1.18 (0.05) |
| RAN120 | 0.33 (0.03) | 1.27 (0.11) | 0.76 (0.02) | 1.22 (0.03) | 0.53 (0.02) | 1.20 (0.05) |
| RAN140 | 0.33 (0.03) | 1.29 (0.11) | 0.77 (0.02) | 1.23 (0.03) | 0.53 (0.02) | 1.21 (0.05) |
| RAN160 | 0.33 (0.03) | 1.29 (0.11) | 0.77 (0.02) | 1.24 (0.03) | 0.54 (0.02) | 1.24 (0.05) |
| RAN180 | 0.34 (0.03) | 1.30 (0.11) | 0.77 (0.02) | 1.24 (0.03) | 0.55 (0.02) | 1.26 (0.05) |

^1^ Bias is presented as the expected regression.

^2^ DM = dry matter yield; HD = heading date; RUST = rust resistant; FILTLOW = strategy filtering out SNPs having low average depth; FILTHIGH = strategy filtering out SNPs having high average depth; FILTBOTH = strategy filtering out SNPs having both low average and high average depth; RAN = strategy keeping SNPs randomly with different data size.
